# Full-colour 3D holographic augmented-reality displays with metasurface waveguides

**DOI:** 10.1038/s41586-024-07386-0

**Published:** 2024-05-08

**Authors:** Manu Gopakumar, Gun-Yeal Lee, Suyeon Choi, Brian Chao, Yifan Peng, Jonghyun Kim, Gordon Wetzstein

**Affiliations:** 1 Department of Electrical Engineering, Stanford University, Stanford, CA USA; 2 Department of Electrical and Electronic Engineering, The University of Hong Kong, Hong Kong, China; 3 NVIDIA, Santa Clara, CA USA

**Keywords:** Displays, Nanophotonics and plasmonics

## Abstract

Emerging spatial computing systems seamlessly superimpose digital information on the physical environment observed by a user, enabling transformative experiences across various domains, such as entertainment, education, communication and training^1–3^. However, the widespread adoption of augmented-reality (AR) displays has been limited due to the bulky projection optics of their light engines and their inability to accurately portray three-dimensional (3D) depth cues for virtual content, among other factors^4,5^. Here we introduce a holographic AR system that overcomes these challenges using a unique combination of inverse-designed full-colour metasurface gratings, a compact dispersion-compensating waveguide geometry and artificial-intelligence-driven holography algorithms. These elements are co-designed to eliminate the need for bulky collimation optics between the spatial light modulator and the waveguide and to present vibrant, full-colour, 3D AR content in a compact device form factor. To deliver unprecedented visual quality with our prototype, we develop an innovative image formation model that combines a physically accurate waveguide model with learned components that are automatically calibrated using camera feedback. Our unique co-design of a nanophotonic metasurface waveguide and artificial-intelligence-driven holographic algorithms represents a significant advancement in creating visually compelling 3D AR experiences in a compact wearable device.

## Abstract (web summary)

We develop a method for providing high-quality, holographic, three-dimensional augmented-reality images in a small form factor suitable for incorporation in eyeglass-scale wearables, using high-refraction-index glass waveguides with nanoscale metasurfaces, and incorporating artificial intelligence.

## Main

**Fig. 1:**
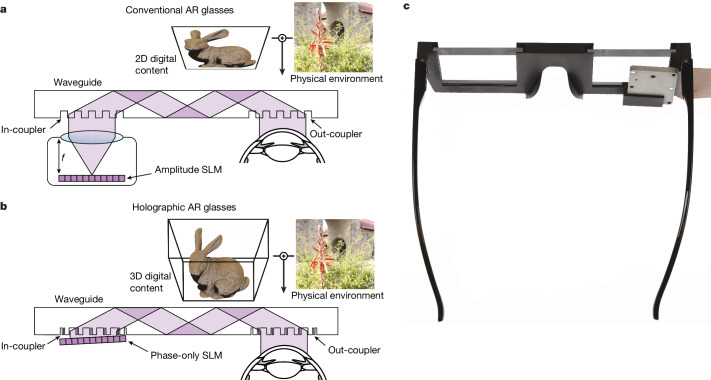
Illustration of the optical principle of waveguide-based AR displays. **a**, Conventional AR glasses use amplitude SLMs, such as organic light-emitting diodes or micro light-emitting diodes, which require a projector-based light engine that is typically at least as thick as the focal length *f* of the projection lens. **b**, The design of our holographic AR glasses uses a phase-only SLM that can be mounted very close to the in-coupling grating, thereby minimizing the device form factor. Additionally, unlike conventional AR glasses, our holographic design can provide full 3D depth cues for virtual content, as illustrated by the bunny (adapted from the Stanford Computer Graphics Laboratory). **c**, Compact 3D-printed prototype illustrating the components of our holographic AR glasses in a wearable form factor.

Emerging augmented-reality (AR) systems offer new experiences to users and have far-reaching implications for applications that span entertainment, education, communication, training, behavioural therapy and basic vision research^1–3^. To unlock their full potential in consumer applications, however, AR display systems must be compact—ideally no larger than conventional eyeglasses—to enable comfort and style for all-day use. Among the plethora of optical designs proposed for such near-eye displays^6,7^, waveguide image combiners are the most promising solution for AR glasses because of their compact form factors. Current waveguide designs, however, require projection optics with a thickness proportional to the focal length of the projection lens (Fig. 1a), introducing optical bulk, and they are limited to displaying two-dimensional (2D) images at a fixed distance to the user. These limitations result in reduced perceptual realism and visual discomfort due to the vergence–accommodation conflict^4,5^ and, even with small projector optics, it is challenging to achieve a device form factor that matches the style of common eyeglasses.

Holographic principles^8^ could enable the ‘ultimate display’^9^ using their ability to produce perceptually realistic 3D content using ultrathin optical films^10,11^. This ability motivated previous attempts to adapt digital holography to AR display configurations^12,13^; though promising, these methods failed to achieve the compact form factors and high 3D image quality required to unlock future spatial computing applications.

Here we develop a new AR display system that pairs a lensless holographic light engine with a metasurface waveguide optimized for full-colour optical-see-through (OST) AR display applications in a compact form factor (Fig. 1b). Compared with other waveguides, our optical system is unique in enabling the relay of full-colour 3D holographic images with high uniformity and see-through efficiency. This remarkable capability is enabled by the use of inverse-designed metasurface^14–16^ grating couplers. Metasurfaces^17,18^ have been demonstrated to offer higher diffraction efficiency^19^, spectral selectivity^20^, *Q*-factor^21^ and transmittance^22^ than conventional refractive and diffractive optical elements in applications, including AR^23^, virtual reality^24^ and wearable devices^20^. Unlike these approaches, ours not only optimizes the devices and demonstrates novel applications of metasurfaces, but also co-designs the entire optical system, including the geometry of a high-index glass waveguide and the metasurface grating couplers, to enable compatability with holographic AR display systems. Waveguide holography has been described in recent work for non-see-through virtual reality settings^25^, but it has seen limited adoption because of its poor image quality. To address this challenge, we develop a mathematical model that describes the propagation of coherent waves in a waveguide using a combination of physically accurate modelling techniques and artificial intelligence. The learnable parts of this model are automatically calibrated using camera feedback with our prototype. This approach significantly advances recent artificial-intelligence-driven holography algorithms^26–29^ by making them suitable for compact waveguides in see-through AR configurations. With our system, we obtained high-quality, full-colour multiplane 3D holographic images using a single OST AR waveguide. Compared with related optical designs^30–33^, our system provides unprecedented full-colour image quality in a compact form factor, enabling a path towards true 3D holographic AR glasses.

## Inverse-designed metasurface waveguide

For OST AR displays, it is critical to provide the user with an unobstructed view of the physical environment while overlaying digital information on their vision of the world. Waveguide image combiners are thin transparent optical systems that have become the industry norm for these applications^7^, enabling the aforementioned capabilities. Our metasurface waveguide system design optimizes compactness, dispersion correction, transmission efficiency and angular uniformity to meet the high demands of 3D-capable AR applications.

**Fig. 2:**
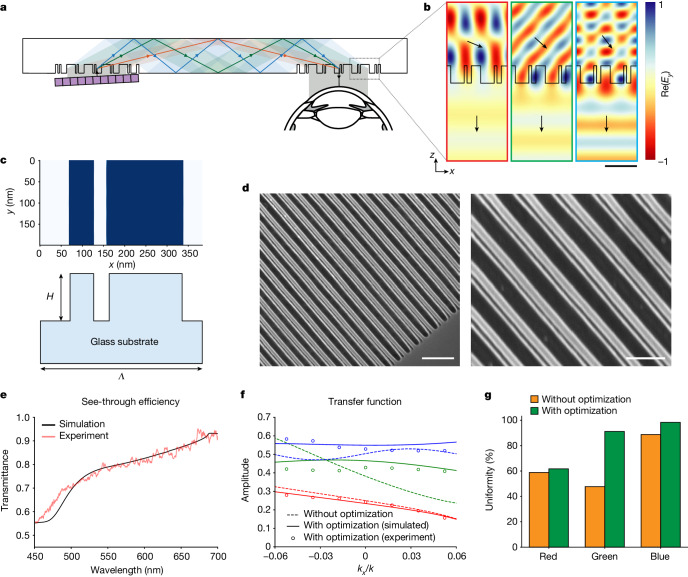
Design and evaluation of our inverse-designed metasurfaces. **a**, Visualization of the waveguide geometry for full-colour operation. **b**, Electric field maps at red (638 nm), green (521 nm) and blue (445 nm) wavelengths for light passing through the metasurface out-coupler towards the user’s eye. The black arrows illustrate the wave vectors of the incident and diffracted light. **c**, Visualization of the inverse-designed metasurfaces optimized for waveguide couplers. The period (*Λ*) and height (*H*) of the metasurfaces are 384 nm and 220 nm, respectively. **d**, Scanning electron microscope images of the fabricated metasurfaces. **e**, The simulated and experimentally measured transmittance spectra of unpolarized light for the inverse-designed metasurfaces in the visible range, corresponding to see-through efficiency for real-world scenes. **f**, The simulated (dashed lines) transfer functions along the *x* axis for the conventional single-lined gratings and the simulated (solid lines) and experimentally measured (circles) transfer functions for our inverse-designed metasurfaces. The colour of the plots corresponds to the red, green and blue wavelengths. The designed metasurfaces are much more efficient than conventional gratings in green and blue, but, due to the very large diffraction angle of red, further improvement of the efficiency of the red channel is more difficult. **g**, Uniformities of the transfer functions for the conventional gratings without optimization and the inverse-designed metasurfaces with optimization. Scale bars, 400 nm (**b**), 2 μm (**d**, left), 200 nm (**d**, right). *E*, electromagnetic field.

Precise manipulation of coherent wavefronts in a waveguide system is crucial for holographic displays, but is very challenging due to the interfering nature of coherent light. We address this challenge using a high-index glass material with a homogeneous design of all-glass metasurfaces (Fig. 2). For a compact waveguide system to minimize boundary reflection and interference, a single-layer coupler is necessary. This coupler must guide broadband visible light through the waveguide at a high diffraction angle, ensuring total internal reflection (TIR). The critical angle, represented as ${\theta }_{{\rm{c}}}(\lambda )={\sin }^{-1}\left(\frac{1}{n(\lambda )}\right)$, dictates that shorter wavelengths *λ* require a higher refractive index *n* to achieve TIR. Our numerical analysis indicates that a refractive index of 1.8 or higher is necessary to transmit all red, green and blue wavelengths through a single coupler, with a higher index expanding the field of view. This underscores the importance of employing a high-index material in our system design. In addition, the high-index glass (*n* > 1.8), with a complex refractive index denoted as $\widetilde{n}=n+ik$, assures minimal absorption loss (*k* ≈ 0) and provides sufficient light–matter interaction, while typical glass (*n* < 1.5) is insufficient to locally manipulate electromagnetic waves due to weak light–matter interaction. As a result, the high-index glass metasurface attains a balance between high see-through efficiency and diffraction efficiency, surpassing the capabilities of typical glass metasurfaces.

Although the high-index glass enables propagation of broadband light with TIR, dispersion correction is further required for full-colour operation. Dispersion-engineered metasurfaces could be an option^34,35^, as a device-level solution, but they often have insufficient degrees-of-freedom to meet the system performance required for AR applications (namely, high uniformity and see-through efficiency). To this end, we correct the chromatic dispersion at the system level through geometric design of the metasurface waveguide system and k-vector matching of the input and output couplers. The in- and out-couplers are designed to have the same momentum but with an opposite direction, so they can couple the incident light in and out without observable dispersion.^7^ Additionally, to spatially match the couplers, we design a dispersion-compensating waveguide geometry by precisely engineering the waveguide thickness and the dimensions and distances of the symmetric metasurface couplers. The lateral displacement of a replicated pupil inside the waveguide can be expressed as $l(\lambda )=2{d}_{{\rm{w}}{\rm{g}}}\tan \left({\sin }^{-1}(\frac{\lambda }{n(\lambda )\varLambda })\right)$, where *d*_wg_, *λ* and *Λ* are the waveguide thickness, the wavelength of light in free space and the grating period, respectively. Our idea is to design the waveguide geometry to have a suitable least common multiple of the $l\left(\lambda \right)$ function for red, green and blue wavelengths, which can be described by ∃ *d*_wg_, *Λ*: LCM( *l*(*λ*_R_), *l*(*λ*_G_), *l*(*λ*_B_) ) < *L*_wg_, where *L*_wg_ is the maximum length between in- and out-couplers for a compact near-eye display and LCM is the least common multiple function. Specifically, we set *d*_wg_ and *Λ* to 5 mm and 384 nm, respectively; with these parameters, the red, green and blue wavefronts from the in-coupler propagate through the waveguide through one, three and five internal reflections, respectively, before meeting at the out-coupler, as illustrated in Fig. 2a.

To optimize the geometry of the metasurface gratings for maximum diffraction efficiency and uniformity of angular response, we employ a rigorous-coupled-wave-analysis solver^36^. Our metasurface couplers operate in transverse electric polarization mode to provide a more uniform optical response. The optimization process uses the gradient descent method, starting from a randomly initialized geometry in the 2D spatial domain and utilizing the Adam solver^37^ to refine the profiles of the metasurface gratings. The loss function in the optimization loop maximizes the sum of the first diffraction order efficiencies for red, green and blue wavelengths (638 nm, 521 nm and 445 nm), while minimizing the standard deviations of efficiencies for different incident angles, ranging from −5° to 5°, for these three wavelengths. We simplify the design process to one dimension by assuming *x* axis symmetry and account for fabrication tolerances of these large-area metasurfaces by adding Gaussian blur. The resulting design converged to a double-lined metasurface grating, as shown in Fig. 2c. This geometry yields metasurface couplers that steer the incident wave to high diffraction angles for red, green and blue wavelengths, as confirmed by the electric field profiles and overlaid Poynting vectors (Fig. 2b). Importantly, the optimized asymmetric nanostructure not only enhances the diffraction efficiency in one direction but also improves uniformity over the angle of incidence.

Figure 2e shows the high see-through efficiency our inverse-designed metasurface couplers achieve, reaching approximately 78.4% in the visible spectrum. Figure 2f contains the transfer functions of our inverse-designed metasurfaces and typical gratings for red, green and blue wavelengths (full 2D transfer functions are shown in the Supplementary Information). As opposed to conventional gratings, our metasurfaces exhibit uniform transmittance regardless of the angle of incidence, thanks to the optimized electromagnetic resonances in the nanostructures. Figure 2g quantifies the uniformity of the transfer function that is defined as the ratio of the minimum and maximum amplitudes within the viewing angle range. The inverse-designed metasurface has high uniformities of 61.7%, 91.2% and 98.3% for red, green and blue, respectively, whereas conventional gratings achieve much lower uniformities of 58.9%, 47.7% and 88.8%. These findings confirm that our inverse-designed all-glass metasurface couplers provide excellent angular uniformity and high see-through efficiency for full-colour operation.

A key challenge for the fabrication of holographic waveguides is a high sensitivity to surface irregularities or particle contamination, which directly affects the observed image quality. For this reason, we fabricate our metasurface system directly on lead-containing high-index glass (SF6 glass, SCHOTT), without any other composing materials, using electron beam (e-beam) lithography. To avoid residue particle contamination or surface damage of the lift-off process or surface irregularities introduced by physical etching, we avoid commonly used lithography processes for metasurface fabrication, including positive e-beam resist with metal lift-off or negative e-beam resist to make an etching mask. Instead, our method is based on reverse patterning with a positive e-beam resist (polymethyl methacrylate (PMMA)) using multiple dry etching methods, thus avoiding lift-off hard masks and ensuring the glass surface remains protected throughout the fabrication process (Methods). Note that this method can also be applied to photolithography or nanoimprint lithography for mass production^38,39^.

## Waveguide propagation model

To simulate the propagation of coherent light through our metasurface waveguide, we first derive a physically motivated model. We then show how this model can be parameterized by neural network components that can be automatically learned from camera feedback. As shown by our experiments, the unique combination of physical and artificial-intelligence components is crucial for accurately modelling the physical optics of such a waveguide and synthesizing high-quality holograms with it.

The wavefront *u*_IC_ coupled into the waveguide can be computed as the product of the phase-only spatial light modulator (SLM) pattern, e^i*ϕ*^, the incident illumination and the in-coupler aperture *a*_IC_. Since we use a converging wavefront for illumination with focal length *f*_illum_, the in-coupled wavefront is expressed as

1 $${u}_{{\rm{I}}{\rm{C}}}({{\rm{e}}}^{{\rm{i}}\phi })={{\rm{e}}}^{-{\rm{i}}\frac{2\pi }{\lambda }\sqrt{{x}^{2}+{y}^{2}+{f}_{{\rm{i}}{\rm{l}}{\rm{l}}{\rm{u}}{\rm{m}}}^{2}}}{{\rm{e}}}^{{\rm{i}}\phi }{a}_{{\rm{I}}{\rm{C}}},$$

where and *x* and *y* are the transverse coordinates.

Next, this wavefront is propagated through the waveguide to compute the out-coupled field, *u*_OC_. A physically motivated model of the waveguide is adequately described by its frequency-dependent transfer function, *H*_WG_ and the aperture *a*_OC_ of the out-coupler:

2 $${u}_{{\rm{O}}{\rm{C}}}({{\rm{e}}}^{{\rm{i}}\phi })={a}_{{\rm{O}}{\rm{C}}}\iint {\mathcal{F}}({u}_{{\rm{I}}{\rm{C}}}({{\rm{e}}}^{{\rm{i}}\phi })){H}_{{\rm{W}}{\rm{G}}}{{\rm{e}}}^{{\rm{i}}2\pi ({f}_{x}x+{f}_{y}y)}{\rm{d}}{f}_{x}{\rm{d}}{f}_{y},$$

where ${\mathcal{F}}$ is the Fourier transform and *f*_x_ and *f*_y_ are the frequency coordinates. The transfer function *H*_WG_ incorporates the reflection coefficients within the waveguide, coupling efficiencies, the propagation of the first diffracted order and the translation between the in- and out-coupler. The contributions of each of these components are used to derive the full expression for *H*_WG_ in our Supplementary Information. Note that we can set *H*_WG_ to the identity operator, ignoring the transfer function, as a naive, non-physical baseline.

Finally, the 3D images observed by a user looking through the holographic AR glasses can be simulated by propagating the out-coupled field with a model of free-space propagation, *f*_free_, to different target distances, *d*_target_, in front of the viewer:

3 $${f}_{{\rm{WG}}}\left({{\rm{e}}}^{{\rm{i}}\phi },{d}_{{\rm{target}}}\right)={f}_{{\rm{free}}}\left({u}_{{\rm{OC}}}({{\rm{e}}}^{{\rm{i}}\phi }),{d}_{{\rm{target}}}\right).$$

With these equations, *f*_WG_ maps phase patterns shown on the SLM to the image that a user would see while focusing at a particular depth, *d*_target_, through the waveguide, and *f*_free_ maps the wavefront in front of the user’s eye to the image that a user would see while focusing at a particular depth, *d*_target_.

**Fig. 3:**
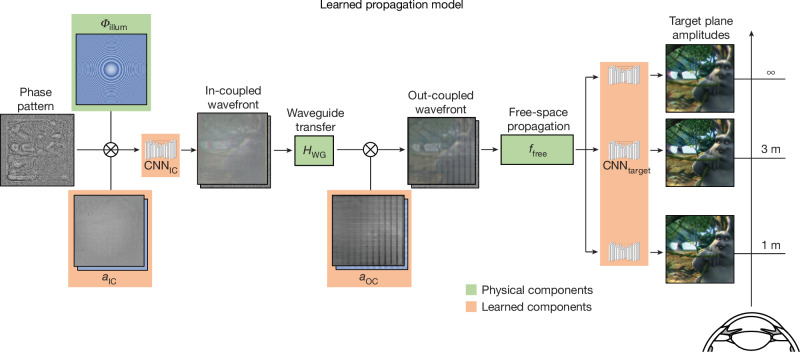
Illustration of the proposed wave propagation model. We combine physical aspects of the waveguide (highlighted in green) with artificial-intelligence components that are learned from camera feedback (highlighted in orange). In our model, the input phase pattern (left) applies a per-pixel phase delay, from 0 to 2π, to the converging illumination before the wavefront is modulated by the learned in-coupler efficiency. This wavefront is then sent through a CNN at the in-coupler plane and propagated through the waveguide, using its physically motivated transfer function, before an additional learned out-coupler efficiency is used to determine the out-coupled wavefront (centre). The latter is propagated to the target scene at various distances from the user where a CNN is applied, converting the complex-valued field into observed intensities (right). When trained on a captured dataset, the learned parameters of the CNNs, the coupler efficiencies and the waveguide propagation enable this model to accurately predict the output of our holographic AR glasses. The model is fully differentiable, enabling simple gradient descent CGH algorithms to compute the phase pattern for a target scene at runtime. The bunny scene is from *Big Buck Bunny*, © 2008 Blender Foundation/www.bigbuckbunny.org, under a Creative Commons licence CC BY 3.0.

Although a physical model, such as *f*_WG_, should accurately describe the wave propagation in a waveguide, in practice it is challenging to model all aspects of such a physical optical system at the required accuracy. Nanoscopic differences, on the order of the wavelength of light, between the simulated model and the optical aberrations, fabrication errors, source beam, or electro-optical effect of the SLM strongly degrade the observed holographic image quality. To account for these small differences between the simulated model and physical optics, we add learnable components in the form of convolutional neural networks (CNNs) to our model. Although related approaches have recently been proposed for bulky benchtop holographic virtual reality displays^26,40–42^, ours characterizes the propagation of full-colour coherent wavefronts through an OST waveguide using this emerging paradigm. Specifically, we propose to learn parameters *a*_IC_ and *a*_OC_ as complex-valued fields, the spatially varying diffraction efficiencies and the CNNs at the in-coupler and target planes to account for a mismatch between simulated model and physical optics. These learned components, which are illustrated with our full waveguide model in Fig. 3, result in the following learnable physical waveguide model:

4 $$\begin{array}{c}\,\,{u}_{{\rm{I}}{\rm{C}}}({{\rm{e}}}^{{\rm{i}}\phi })={{\rm{C}}{\rm{N}}{\rm{N}}}_{{\rm{I}}{\rm{C}}}({{\rm{e}}}^{-{\rm{i}}\frac{2\pi }{\lambda }\sqrt{{x}^{2}+{y}^{2}+{f}_{{\rm{i}}{\rm{l}}{\rm{l}}{\rm{u}}{\rm{m}}}^{2}}}{{\rm{e}}}^{{\rm{i}}\phi }{a}_{{\rm{I}}{\rm{C}}})\\ {f}_{{\rm{W}}{\rm{G}}}({{\rm{e}}}^{{\rm{i}}\phi },{d}_{{\rm{t}}{\rm{a}}{\rm{r}}{\rm{g}}{\rm{e}}{\rm{t}}})={{\rm{C}}{\rm{N}}{\rm{N}}}_{{\rm{t}}{\rm{a}}{\rm{r}}{\rm{g}}{\rm{e}}{\rm{t}}}({f}_{{\rm{f}}{\rm{r}}{\rm{e}}{\rm{e}}}({u}_{{\rm{O}}{\rm{C}}}({{\rm{e}}}^{{\rm{i}}\phi }),{d}_{{\rm{t}}{\rm{a}}{\rm{r}}{\rm{g}}{\rm{e}}{\rm{t}}})).\end{array}$$

In Methods, we detail our training procedure and CNN architecture.

## Experimental results

Our prototype AR display combines the fabricated metasurface waveguide with a HOLOEYE LETO-3 phase-only SLM. This SLM has a resolution of 1080 × 1920 pixels with a pitch of 6.4 μm. A FISBA READYBeam fibre-coupled module with optically aligned red, green and blue laser diodes with wavelengths of 638, 521 and 445 nm is used as the light source. Since our illumination comes through the back of our waveguide, we slightly tilt our SLM and illumination, so that our digital content is not obscured by any unwanted light that is coupled into the waveguide before reaching the SLM. We capture calibration data for our artificial-intelligence-based wave propagation model and also capture results of using a FLIR Grasshopper3 12.3 MP colour USB3 sensor through a Canon EF 35 mm lens with an Arduino controlling the focus of the lens. Following recent work^42^, our experimental setup operates in a partially coherent setting where a few coherent modes are multiplexed in time to achieve optimal 3D holographic image quality with realistic depth-of-field effects. All holograms are computed using a gradient descent computer-generated holography (CGH) algorithm^26^ that incorporates our camera-calibrated wave propagation model.

**Fig. 4:**
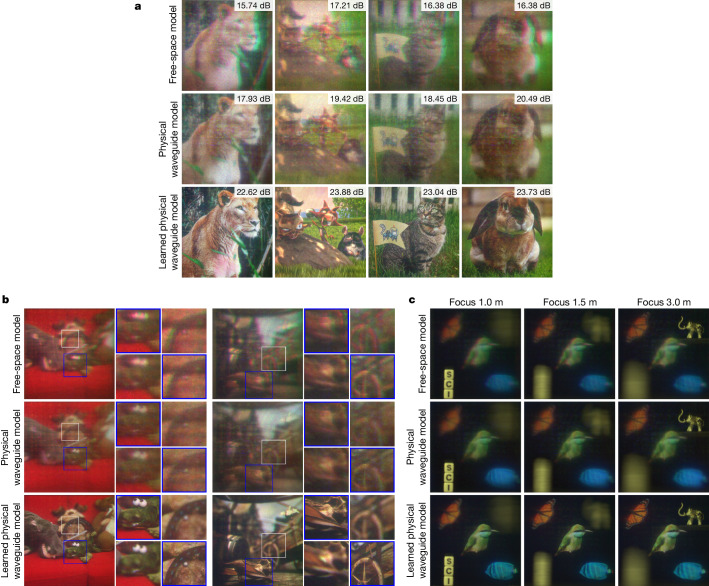
Experimental results captured through our compact holographic display prototype. **a**, Comparison of 2D holograms synthesized using several different wave propagation models, including free-space propagation, a physically motivated model and our proposed model combining physics and learnable parameters that are calibrated using camera feedback. **b**, Comparison of two 3D holograms. Zoomed-in crops show the scene with the camera focused at different depths. Blue boxes highlight content that the camera is focused on while white boxes emphasize camera defocus. **c**, Comparison of a 3D hologram captured in an optical-see-through AR mode. The bird, fish and butterfly are digitally superimposed objects, and the elephant and letters are part of the physical environment. In all examples, the proposed wave propagation model represents the physical optics much more accurately, resulting in significant image quality improvements over alternative models. In **a**, the squirrel scene is from *Big Buck Bunny*, © 2008 Blender Foundation/www.bigbuckbunny.org, under a Creative Commons licence CC BY 3.0. In **b**, couch and market target scenes are, respectively, from the High Spatio-Angular Light Field dataset^49^ and the Durian Open Movie project (© copyright Blender Foundation/durian.blender.org) under a Creative Commons licence CC BY 3.0.

We show experimentally captured results from our prototype in Fig. 4. In Fig. 4a, we qualitatively and quantitatively assess the 2D image quality and compare a naive free-space propagation model, a physically motivated wave propagation model using the rigorous-coupled-wave-analysis-simulated transfer functions and the proposed artificial-intelligence-based variant combining the physical model with camera-calibrated learnable parameters. In all examples, the artificial-intelligence-based wave propagation model outperforms the baselines by a large margin of 3–5 dB peak signal-to-noise ratio. The full-colour 3D results shown in Fig. 4b validate the high image quality our system achieves for both in- and out-of-focus regions of the presented digital content. The accurate depiction of 3D defocus behaviour can mitigate the vergence–accommodation conflict and associated discomfort for users of our display system. To our knowledge, no existing waveguide-based AR display has demonstrated full-colour 3D results with a comparable quality^25,43^. Finally, we also show experimental full-colour 3D results in Fig. 4c where we optically combine a physical scene with digitally overlaid content and capture the scene using different focus settings of the camera. Again, our approach outperforms baseline models by a large margin.

## Discussion

The co-design of a metasurface waveguide and artificial-intelligence-based holography algorithms facilitates a compact full-colour 3D holographic OST AR display system. To our knowledge, no system with comparable characteristics has previously been described and our experimental image quality far exceeds that demonstrated by related waveguide designs for non-see-through applications^25^.

The field of view of our waveguide design is currently limited to 11.7°. While this is comparable to many commercial AR systems, it would be desirable to enlarge it. This could be achieved using higher refractive index materials for the waveguide or by engineering an additional metasurface eyepiece into the out-coupler. Related ideas have recently been explored for other optical AR system designs^23^, which could be adapted to ours. Our waveguide is compact, but it would be interesting to further reduce its thickness *d*_wg_. In our Supplementary Information, we derive the relationship between waveguide thickness, SLM size *L*_slm_ and nasal field of view *θ*_−_ as

5 $${d}_{{\rm{wg}}}\ge \frac{{L}_{{\rm{slm}}}}{2\tan \left({\sin }^{-1}\left[\left(\frac{{\lambda }_{{\rm{B}}}}{\Lambda }-\sin ({\theta }_{-})\right)\frac{1}{n({\lambda }_{{\rm{B}}})}\right]\right)}.$$

This equation shows that the thickness of the waveguide is directly proportional to the SLM size, among other factors. Therefore, the most promising path to reducing the thickness of the waveguide is to use a smaller SLM. There is a clear path to achieving this with emerging SLMs that provide very small pixel pitches, down to 1 μm (ref. ^44^), compared with the 6.4 μm of our SLM. Although not commercially available yet, these SLMs would enable ultrathin waveguides using our approach.

Similar to all holographic displays, the étendue of our display is limited by the space–bandwidth product of the SLM. Étendue expansion techniques^7,43,45–47^ could be adapted to our settings, although no such technique has been demonstrated to support full-colour 3D waveguide holography. Another potential direction for future work would be to combine our design with an illumination waveguide as shown in prior work for a compact illumination path^25^. Finally, we have not attempted to optimize the efficiency of our CGH algorithm at runtime. While hologram generation currently takes several minutes per phase pattern, recent methods have shown that real-time inversion of wave propagation models for hologram synthesis can be achieved using machine-learning approaches^26,27,29,48^.

The proposed co-design of nanophotonic hardware and artificial-intelligence-driven algorithms enables optical-see-through AR display modes in smaller form factors and with higher 3D image quality than any existing approach of which we are aware, enabling a path towards true 3D holographic AR glasses.

## Methods

### Fabrication details

The fabrication procedure begins by coating the substrate with a 30-nm-thick Chromium (Cr) film through e-beam evaporation (Kurt J. Lesker Company). We then proceed to an e-beam lithography process (Raith Voyager) using a 50 kV e-beam to accurately create the metasurface patterns with a dimension of 6.5 mm by 6.5 mm for the in-coupler and 6.5 mm by 7.1 mm for the out-coupler, after spin-coating a positive-tone e-beam resist layer (950 PMMA a4, 1000 rpm for 60 s), post-backing the PMMA layer (180 °C for 5 min) and spin-coating a charge dissipation layer (e-spacer, Showa Denko). Then the patterns are transferred onto the high-index glass substrate using multiple dry etching steps. These steps involve an inductively coupled plasma reactive ion etcher (ICP-RIE, PlasmaTherm Metal Etcher) for Cr etching with the PMMA mask and a reactive ion etcher (RIE, Oxford Dielectric Etcher) for glass etching with the Cr mask, with a specific gas mixture of Cl_2_, O_2_, CHF_3_, CF_4_ and Ar, and further aided by helium backside cooling. The remaining Cr mask is eliminated by an additional ICP-RIE process. Figure 2d presents the scanning electron microscope images of the precisely fabricated all-glass metasurface couplers.

Metasurface sample images are taken by a scanning electron microscope (FEI Nova NanoSEM 450). The representative samples are coated with a thin 3 nm film of gold/palladium to reduce charing in the images. Images are acquired with an accelerating voltage of 10 kV.

### CNN network architecture

Our CNNs, CNN_IC_ and CNN_target_, use a modified UNet architecture^50^ to efficiently learn the residual aberrations in a physical optical system. The input wavefront is augmented by concatenating its real and imaginary values with their corresponding amplitude and phase components. After the input layer, both CNNs use 32 feature channels and perform five downsampling operations using strided convolutions, as well as five upsampling operations using transposed convolutions. The networks use instance normalization^51^, leaky rectified linear unit activation (slope −0.2) for the down blocks, rectified linear unit nonlinearities for the up blocks and skip connections. CNN_IC_ has two-channel outputs representing the real and imaginary values, while CNN_target_ directly outputs a single-channel amplitude. *a*_IC_ and *a*_OC_ are the binary aperture functions of the grating couplers for the physically motivated wave propagation model. When using the artificial-intelligence-augmented model, these quantities are complex-valued fields that are learned per colour channel.

### Training the waveguide model

We train our neural-network-parameterized wave propagation model using a dataset comprising a large number of pairs of SLM phase patterns and corresponding intensity images captured by a camera focusing at different depths at the output of our prototype holographic display. The SLM phase patterns in our dataset are generated using our physical waveguide model to produce images from the DIV2K dataset, at different virtual distances through the waveguide. The model is trained over four intensity planes, corresponding to 0 D (*∞* m), 0.33 D (3 m), 0.67 D (1.5 m), 1.0 D (1 m) in the physical space. We perform our model training on a 48 GB NVIDIA RTX A6000 with a batch size of 1 and a learning rate of 3 × 10^−4^. We note that the diversity of phase patterns is important for the model training. A dataset generated using the gradient descent CGH algorithm^26^ typically consists of holographic images that primarily cover a narrow angular spectrum. Thus, we generate phase patterns with a set of random parameters, including learning rates, initial phase distribution and propagation distances. We generate 10,000 patterns for each channel and capture the corresponding intensities. The dataset is divided into training, validation and test sets with a ratio of 8:1:1. The initially trained model can be used to synthesize an additional phase dataset that is used to refine the model. Such a refinement stage improves the experimental quality. We perform this refinement procedure twice for the best quality. After this training procedure, we use our learned waveguide propagation model to synthesize holograms for new 2D and 3D scenes enabling our holographic AR glasses to operate without any additional camera feedback.

## Online content

Any methods, additional references, Nature Portfolio reporting summaries, source data, extended data, supplementary information, acknowledgements, peer review information; details of author contributions and competing interests; and statements of data and code availability are available at 10.1038/s41586-024-07386-0.

### Supplementary information

Supplementary Information This file contains Supplementary Notes 1–5, Figs. 1–18, Table 1 and References.

Supplementary Video 1 Laser-synchronized 2D video results, 3D video results, 2D AR video results and 3D AR video results.

Supplementary Video 2 Metasurface optimization animation.

## Data Availability

A full-colour captured dataset specific to our holographic AR glasses prototype is available upon request.

## Code availability

Computer code supporting the findings of this study is available online at https://github.com/computational-imaging/holographic-AR-glasses.git.
