# Supplementary material for: Full-colour 3D holographic augmented-reality displays with metasurface waveguides
Source: Nature. 2024 May 8;629(8013):791–7. doi: 10.1038/s41586-024-07386-0 (PMC11111399; doi:10.1038/s41586-024-07386-0)
Supplement: Supplementary file 1 — This file contains Supplementary Notes 1–5, Figs. 1–18, Table 1 and References. [file 41586_2024_7386_MOESM1_ESM.pdf]

nature portfolio

https://doi.org/10.1038/s41586-024-07386-0

Supplementary information

Full-colour 3D holographic augmented-reality displays with metasurface waveguides

In the format provided by the authors and unedited

Supplementary Information for

Full-color 3D holographic augmented reality displays with metasurface waveguides

Manu Gopakumar,[1,4] Gun-Yeal Lee,[1,4] Suyeon Choi,[1] Brian Chao,[1] Yifan Peng,[2] Jonghyun Kim,[3] Gordon Wetzstein[1*]

This PDF file includes:

Supplementary Note 1: Additional details on system implementation
Supplementary Note 2: Additional details on metasurface waveguide
Supplementary Note 3: Additional details on fabrication
Supplementary Note 4: Additional details on waveguide model
Supplementary Note 5: Additional experimental results
Supplementary References

[1]Department of Electrical Engineering, Stanford University, Stanford, CA
[2]Department of Electrical and Electronic Engineering, The University of Hong Kong, Hong Kong
[3]NVIDIA, Santa Clara, CA
[4]These authors contributed equally: Manu Gopakumar and Gun-Yeal Lee
*Corresponding author. E-mail: gordon.wetzstein@stanford.edu

Supplementary Note 1: Additional details on system implementation

For our prototype, we use a FISBA READYBeam fiber-coupled module with three optically aligned laser diodes with measured wavelengths of 638.35 nm, 521.16 nm, and 444.50 nm for red, green, and blue respectively for our light source. To produce our converging illumination, we linearly polarize and reimage this light source to a focal point 85.6 mm behind our Holoeye LETO3 phase-only liquid crystal on silicon (LCoS) SLM. The use of converging illumination in our setup magnifies the holographic content produced by our SLM and replaces the eyepiece positioned after the SLM in many recent holographic display works.[1–3] This illumination is directed into the benchtop implementation of our compact holographic AR glasses with a mirror as shown in the labelled photograph of our prototype in Supplementary Figure 1. The LETO3 SLM in our setup has a resolution of 1080 $\times$ 1920 pixels and a pixel pitch of 6.4 μm. After the out-coupler, we have an additional linear polarizer to clean up the polarization after the waveguide. Based on the focal length of the converging illumination and diffraction angle of our SLM, our holographic display can cover a maximum diagonal FOV of 11.83 degrees and support an eyebox of 5.9 mm by 5.9 mm. Compared to prior benchtop setups, this AR prototype shrinks down or removes many components of conventional holographic displays.[1] There is no bulky Fourier filtering in our system which allows us to position our display panel

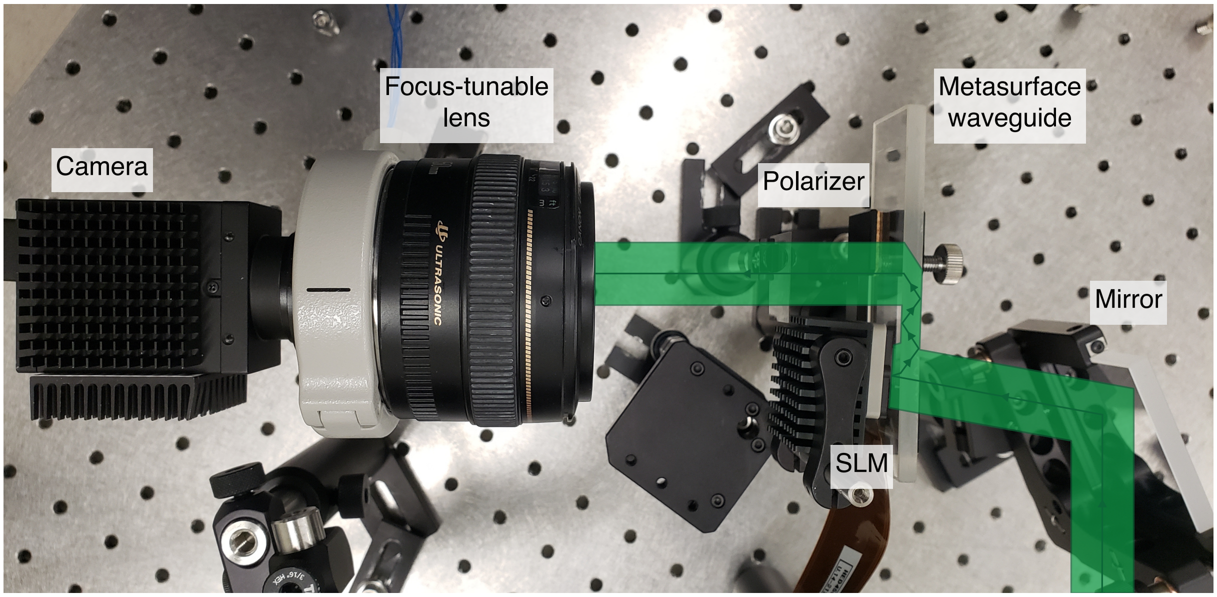

Supplementary Fig. 1 Labelled photograph of our benchtop holographic display with illustrated beam path highlighting the direction of a central ray of light propagating through our system when a blank phase pattern is shown on the SLM.

directly against the waveguide, and the propagation after the SLM is folded into the waveguide. We capture our dataset and results on a benchtop setup using a FLIR Grasshopper3 12.3 MP color USB3 sensor through a Canon EF 35mm lens with an Arduino microcontroller for focus control. For the results in our paper, color images are captured as separate exposures for each wavelength and then merged. To further demonstrate our full color display, we have also included laser-synchronized video holograms with all colors captured simultaneously in the same exposure in our supplementary video (see "Laser-synchronized Video Holograms" section for additional details). Our LCoS phase SLM can display 180 phase patterns per second enabling a 7.5 Hz frame rate for these full-color time-multiplexed holograms, but recently MEMs-based phase SLMs have emerged that can increase this frame rate to 60 Hz for the full-color time-multiplexed holograms.[3] As in prior work,[1] we apply a planar homography from the field of computer vision to align the captured images to the simulated images. Our homography uses a target binary pattern consisting of 10×10 white dots with 80 pixels between the centers of neighboring dots. Accordingly, the aligned region of interest has a resolution of 800×800 pixels.

Along with the compact benchtop prototype, we implement a wearable model of our design shown in our main paper. To validate that our design decisions simultaneously enable compactness and high image quality, we use the same optical configuration for both our benchtop and wearable prototypes. In our wearable design, we use a 3D printed frame to hold our SLM and waveguide together, and the edges of the waveguide are cut down to fit the frame shape. The prototype figures do not include the equipment for driving and illuminating the SLM, and these components would also have to be compactly arranged for a fully functional wearable prototype. While we did not design a compact illumination path in this work, this could be done with an additional illumination waveguide as was illustrated by a prior design for VR holographic glasses.[4]

Supplementary Note 2: Additional details on metasurface waveguide

System parameter analysis

This section discusses a general analysis of the diffraction through our waveguide. With this analysis, we formulate bounds on our system parameters including the waveguide thickness and field of view (FOV).

The diffraction of light in our waveguide can be analyzed with the Generalized Snell's Law[5] at the metasurface couplers, which can be expressed as

$$\sin(\theta_{\text{in}}) + \frac{\lambda}{\Lambda} = n(\lambda)\sin(\theta_{\text{diff}}), \tag{1}$$

where θ_{in} is the incident angle at the coupler, θ_{diff} is the diffracted angle in the waveguide, Λ is the coupler grating period, λ is the wavelength of light in free space, and $n(\lambda)$ is the wavelength-dependent refractive index of our high-index glass plotted in Supplementary Figure 2.

From Eq. 1, we can calculate the diffraction angles of each wavelength across a range of incident angles. For conventional waveguide design, the upper bound for the FOV can be determined based on which of these diffraction angles propagate within the waveguide with total internal reflections (TIR). These angles satisfy two conditions regarding (1) critical angles of TIR and (2) far-field propagation. The TIR condition requires that the minimum diffracted angle is larger than the critical angle. For a minimum incident angle with magnitude, θ_-, this can be expressed as

$$\min\left(\frac{\lambda}{\Lambda} + \sin(\theta_{\text{in}})\right) = \frac{\lambda_B}{\Lambda} - \sin(\theta_-) \geq 1. \tag{2}$$

The far-field propagation condition requires that the maximum diffracted angle is less than 90 degrees, so that reflected waves can propagate within the waveguide without becoming evanescent fields. For a maximum incident angle with magnitude, θ_+, this can be expressed as

$$\max\left(\frac{\lambda}{\Lambda} + \sin(\theta_{\text{in}})\right) = \frac{\lambda_R}{\Lambda} + \sin(\theta_+) \leq n(\lambda_R). \tag{3}$$

For our system, the bounds on the diffracted angles are stricter based on the geometry of the waveguide. Here, the minimum incident angle is further limited by the geometric constraint discussed in the main paper. To have a sufficiently large out-coupler, the lateral displacement between exit pupils for all wavelengths ($l(\lambda)$) should be at least the size of the SLM wavefront (L_{slm}) that is coupled into the waveguide. This constraint, which is necessary to out-couple a single exit pupil, can be expressed as:

$$\min(l(\lambda)) = l(\lambda_B) = 2d_{wg}\tan\left(\sin^{-1}\left[\left(\frac{\lambda_B}{\Lambda} - \sin(\theta_-)\right)\frac{1}{n(\lambda_B)}\right]\right) \geq L_{slm}. \tag{4}$$

Similarly, the maximum incident angle is also further limited for our waveguide by the size of the out-coupler. Specifically the lateral displacement between exit pupils for all wavelengths should be at most the distance across the waveguide (L_{wg}) from one end of the in-coupler to the opposite end of the out-coupler. This constraint can be expressed as:

$$\max(l(\lambda)) = l(\lambda_R) = 2d_{wg} \tan\left(\sin^{-1}\left[\left(\frac{\lambda_R}{\Lambda} + \sin(\theta_+)\right)\frac{1}{n(\lambda_R)}\right]\right) \leq L_{wg}. \tag{5}$$

In these formulas, λ_R and λ_B are the wavelengths of red (638 nm) and blue (445 nm), $n(\lambda_R)$ and $n(\lambda_B)$ are the refractive indices at red (1.798) and blue (1.842), Λ is the grating period (384 nm), L_{slm} is the size of the SLM wavefront (6.5 mm), d_{wg} is the waveguide thickness (5 mm), and L_{wg} is the distance between the far edges of the couplers (30.86 mm).

Field of view of the waveguide

We can rearrange Eqs. 4 and 5 to get the upper bound for the total FOV that our waveguide can support:

$$\begin{aligned}\theta_{FOV} &= \theta_- + \theta_+ \\ &\leq \sin^{-1}\left(\frac{\lambda_B}{\Lambda} - n(\lambda_B)\sin\left[\tan^{-1}\left(\frac{L_{slm}}{2d_{wg}}\right)\right]\right) \\ &\quad + \sin^{-1}\left(n(\lambda_R)\sin\left[\tan^{-1}\left(\frac{L_{wg}}{2d_{wg}}\right)\right] - \frac{\lambda_R}{\Lambda}\right).\end{aligned} \tag{6}$$

When we plug in the parameters for our waveguide, we can see that the upper bound for the FOV of our waveguide is 11.72 degrees.

Relationship between waveguide thickness and SLM size

The lower bound for the thickness of our waveguide can be related to the size of the wavefront from the SLM by rearranging Eq. 4:

$$d_{wg} \geq \frac{L_{slm}}{2\tan\left(\sin^{-1}\left[\left(\frac{\lambda_B}{\Lambda} - \sin(\theta_-)\right)\frac{1}{n(\lambda_B)}\right]\right)} \tag{7}$$

This arrangement shows that the thickness is limited by the size of the wavefront from our SLM. A smaller SLM can enable our waveguide design to be thinner in the future.

Further improvements in field of view

There are a number of promising directions that future works could take to increase the FOV of our waveguide AR system, as discussed in the following.

Integrating a metasurface lens function into the out-coupler can magnify the FOV outputted by our holographic display without requiring the waveguide to support a wider FOV. Prior work has shown that metasurface combiners that do not distort the reality view can be designed to have a lens function, thereby improving the FOV of AR images.[6] However, this approach would shrink the eyebox of our system.

Increasing the FOV of our system without reducing the eyebox can be done by expanding the space–bandwidth product for our holographic display with more SLM pixels. Advanced SLM technology with a better spatial resolution would improve the space–bandwidth product and with it the achievable FOV (and eyebox size). As mentioned in the Discussion section, research grade SLMs have already achieved a $>6\times$ smaller pixel pitch[7] than our commercial SLM. Moreover, recent nanophotonic approaches to develop SLMs with nanoscale pixels based on active metasurfaces or semiconductor monolayer materials could be solutions to further decrease the pixel pitch.[8,9] In this case, the increased FOV would also have to be supported by our waveguide. Since the waveguide system is based on total internal reflection, the refractive index of the waveguide substrate determines the critical angle of total internal reflection, and the higher the refractive index, the higher-angle light can be trapped inside and delivered through the waveguide. In this work, we used a high refractive index glass material (SCHOTT SF6, n $\sim$ 1.8), which not only has superior transparency but also provides a larger FOV for the system than typical glass materials (n $\sim$ 1.5). For AR applications, there is the condition that the waveguide substrate material must be transparent without distorting the real-world scene, so the selection of high refractive index materials is limited. However, recent studies on synthetic materials to obtain optimized refractive index and absorption coefficients could be a promising solution for this. For example, nanoparticle composite-based metasurfaces have been studied using a mixture of high-index nanoparticles and low-index resin materials to obtain an optimized refractive index with better fabrication compatibility, and various refractive index values from 1.5 to 2.1 can be continuously controlled through the weight ratio of high-index TiO_2 nanoparticles.[10] Additionally, very recent metasurface research reported that coating a very thin metal layer on the metasurface nanostructure of a polymer material with a low refractive index amplifies the light-matter interaction, making it possible to obtain an effectively high refractive index value and high diffraction efficiency.[11] In this way, we may be able to achieve a high refractive index material with great transparency, which could be potentially helpful for improving FOV of our waveguide system.

Maximizing the metasurface grating out-coupler range

To determine the maximum valid out-coupler range, we ensure that there is no overlap between unwanted exit pupils and our out-coupler even with the field spread expanding the exit pupils as they propagate through the waveguide. Firstly, the horizontal extent of our in-coupler grating can be denoted as $(0, L_{slm})$, where the origin is defined at the start of the in-coupler. Then, the corresponding horizontal range of our out-coupler grating (OC_{min}, OC_{max}) can be defined. OC_{min} is set to exclude the light from the wavefront that has not reflected within the waveguide enough times, and OC_{max} is set to exclude the light from the wavefront that has reflected within the waveguide too many times. This blocks additional copies of the wavefront from being out-coupled since that would prevent the SLM wavefront from having the degrees of freedom to flexibly control the full out-coupled wavefront. The resulting mathematical expressions for OC_{min} and OC_{max} are below:

$$\begin{aligned} OC_{min} &: d_{wg}\left(N_{TIR}-1\right)\tan\left(\theta_{max}\right)+L_{slm} \\ OC_{max} &: d_{wg}\left(N_{TIR}+3\right)\tan\left(\theta_{min}\right) \end{aligned} \tag{8}$$

where θ_{min} and θ_{max} are the minimum and maximum diffracted angles in the waveguide, and N_{TIR} is the wavelength-dependent desired number of total internal reflections between the in-coupled wavefront and the desired out-coupled exit pupil. Note that, to maximize the valid out-coupler range, we plug in the range of incident and diffracted angles covered by our SLM which is a symmetric subset of the full FOV supported by the waveguide.

To work with three color channels, we set our out-coupler range as the intersection of the out-coupler ranges determined for each color.

$$\begin{aligned} OC_{min} &= \max\left\{OC_{min,red}, OC_{min,green}, OC_{min,blue}\right\} \\ OC_{max} &= \min\left\{OC_{max,red}, OC_{max,green}, OC_{max,blue}\right\} \end{aligned} \tag{9}$$

These equations bound the out-coupler area that we fabricate.

Optimization process of metasurfaces

The simulation and optimization of the inverse-designed metasurface couplers are obtained using the PyTorch-based Rigorous Coupled Wave Analysis (RCWA) solver.[12] The metasurfaces are assumed to have the thickness of 220 nm and the x-axis period of 384 nm. In the RCWA simulation, the simulation space is set to 384 nm by 200 nm in the xy plane in which the x-axis period comes from the waveguide system design and the y-axis period is designed to have no diffraction order in the y-direction and is set small for computational efficiency. Both x- and y-axis have periodic conditions. The refractive indices of high-index glass (SF6 glass, SCHOTT) for the operation wavelengths of red (638 nm), green (521 nm), and blue (445 nm) are 1.798, 1.818, and 1.842, respectively, as presented in Supplementary Figure 2. The see-through efficiency for unpolarized light in Figure 2e is obtained after calculating the zero-order transmission efficiencies for both x- and y-polarized incident light. The transfer functions of the metasurfaces are obtained from calculating the +1 order diffraction efficiencies with respect to incident angles.

For optimization, we set up the RCWA simulation to calculate the first diffraction order for each color, and the 2D geometry of the metasurfaces in the xy plane is updated during iterations. The loss function, L, in the optimization is designed to maximize the first diffraction order efficiency (T_{+1}) for each color, while minimizing the standard deviations (σ) of the diffraction efficiencies for different incident angles (θ), ranging from $-5°$ to $5°$, which can be expressed as

$$L = -\sum_{\lambda}\left(\sum_{\theta} T_{+1}(\rho, \lambda, \theta) - \beta \cdot \sigma_{\theta}(T_{+1}(\rho, \lambda, \theta))\right), \quad (10)$$

where ρ is the 2D geometry of the metasurface and λ is the wavelength of light in free space. The coefficient ($\beta = 1/3$) is the relative weight on the loss components. The learning curve of the loss function is illustrated in Supplementary Figure 3a, and the metasurface geometries for initial, intermediate, and final stages are presented from top to bottom in Supplementary Figure 3b. We also show the evolution of the metasurface geometry throughout the optimization as a supplementary video.

In our optimization, we started from a randomized initial state for the refractive index profile and pushed the optimization process for the inverse-designed metasurface to have a fabrication-friendly profile by the end of the optimization. Since metasurface nanostructures have a constrained index profile (e.g., from 1 for air to the refractive index of the metasurface materials), constraints on the refractive index must be applied to the optimization to obtain a practical or fabrication-feasible metasurface profile. While considering the fabrication feasibility of large-area metasurfaces, it is also important to select the pattern resolution to have sufficient lithography writing speed. The geometric profiles of inverse-designed metasurfaces sometimes produce very challenging profiles that require a few nanometer resolution, which is not practical for real applications with large-area metasurfaces. Therefore, we added constraints to the optimization process for realistic fabrication conditions. Specifically, x-axis symmetry is enforced as there

is no diffraction in the y-axis with a periodic condition, and a Gaussian blur kernel with $\sigma = 20$ nm is applied at every iteration before the optimized refractive index profile is clipped to the feasible refractive indices of air and the metasurface material. These optimization conditions to achieve fabrication feasibility may hinder the solver's ability to converge in a smoothly decaying manner, introducing sudden jumps of the FOM and slowing down the optimization in the early stages, but ultimately this procedure provided us with a fabrication-friendly high performance geometric profile for our metasurface.

Supplementary Figures 4 and 5 show the 2D complex-amplitude transfer functions of the diffraction efficiency of the inverse-designed metasurfaces and conventional grating, respectively. Here the conventional grating is defined as a single-lined grating with a fill factor of 0.5 under the same period, height, and material conditions. These transfer functions correspond to Figure 2f in the main text, showing the uniform diffraction efficiency of our metasurfaces regardless of the incident angle compared to the conventional grating. The 2D complex-amplitude transfer functions are used to build our learned physical waveguide model to account for the optical responses of the in- and out-couplers. Supplementary Figure 6 shows the 2D transfer functions of the see-through efficiency for the inverse-designed metasurface and the conventional grating, illustrating that the see-through transmittance of our metasurfaces is also uniform regardless of the incident angle as opposed to conventional gratings.

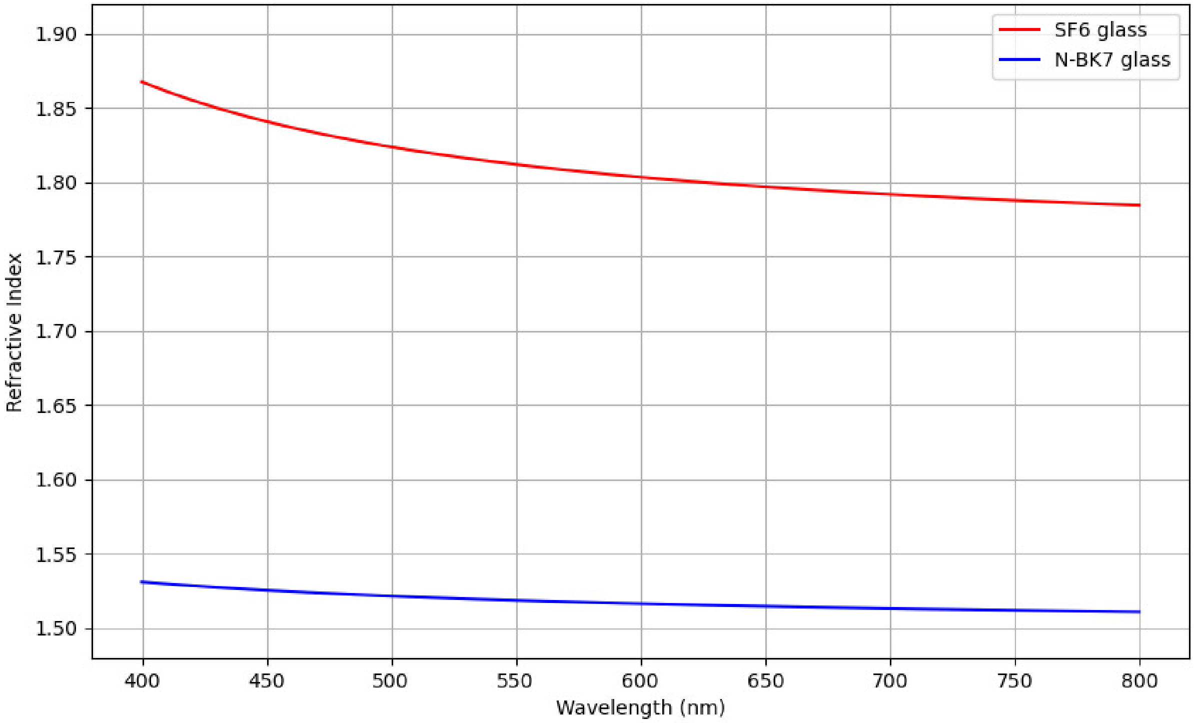

Supplementary Fig. 2 Refractive index profiles for the glass utilized in the prototype fabrication. The plot illustrates the wavelength-dependent refractive index for two types of glass. The red line represents the lead-containing glass used for the fabrication, exhibiting a higher refractive index across the visible spectrum in contrast to typical glass, depicted by the blue line. This higher refractive index allows for total internal reflections at shorter wavelengths, making it particularly suitable for our compact waveguide design. The unique refractive index characteristics of the high-index glass significantly contribute to the effective operation of the metasurface waveguide system.

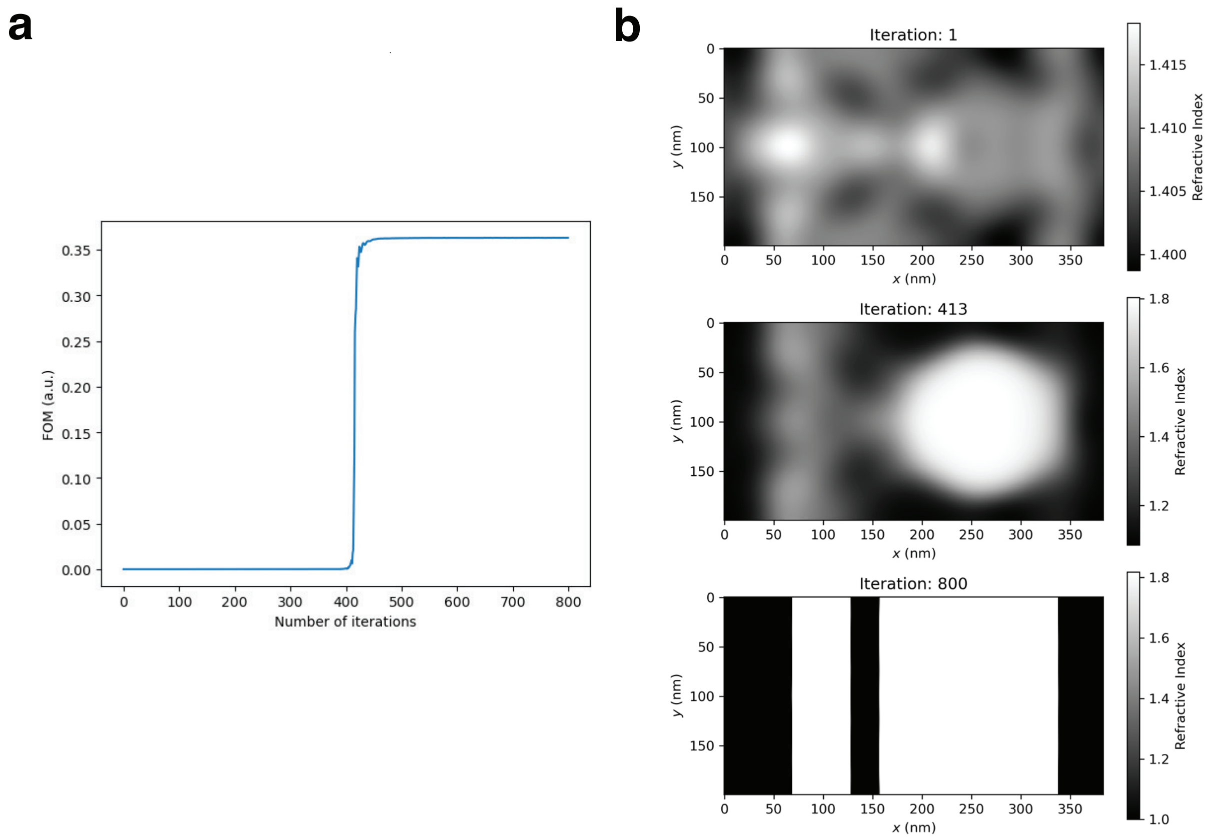

Supplementary Fig. 3 Optimization process of the inverse-designed metasurfaces. (a) Graph depicting the Figure of Merit (FOM) evolution over the course of optimization iterations. The y-axis indicates the FOM, while the x-axis represents the number of iterations. To consider the fabrication feasibility of the electron beam lithography, x-axis symmetry and Gaussian blur are applied in each iteration. (b) Geometry of the metasurfaces in the xy plane at the initial state (top), intermediate state (middle), and after optimization (bottom). The color bar represents the refractive index at a wavelength of 521 nm. These visuals illustrate the effective progression from a random initialization state to a highly optimized metasurface design, evidencing the successful operation of the optimization process.

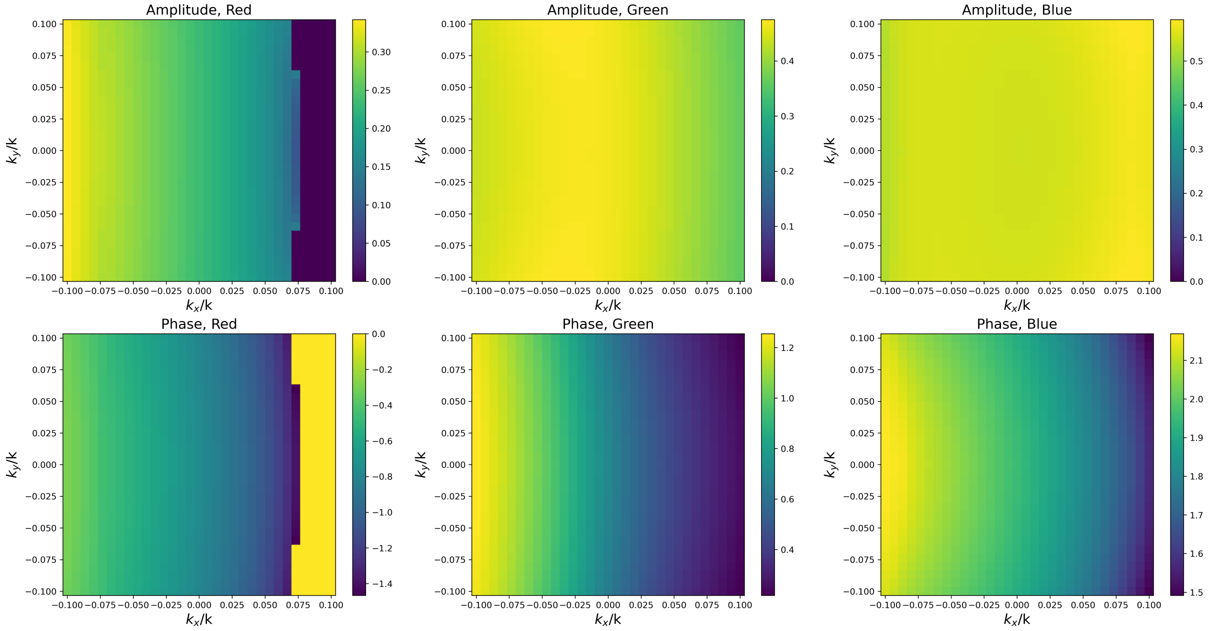

Supplementary Fig. 4 Simulated 2D transfer functions of the diffraction efficiency for the inverse-designed metasurfaces. The amplitude (top) and phase (bottom) of the complex-amplitude transfer functions for the red (left), green (middle), and blue (right) are presented.

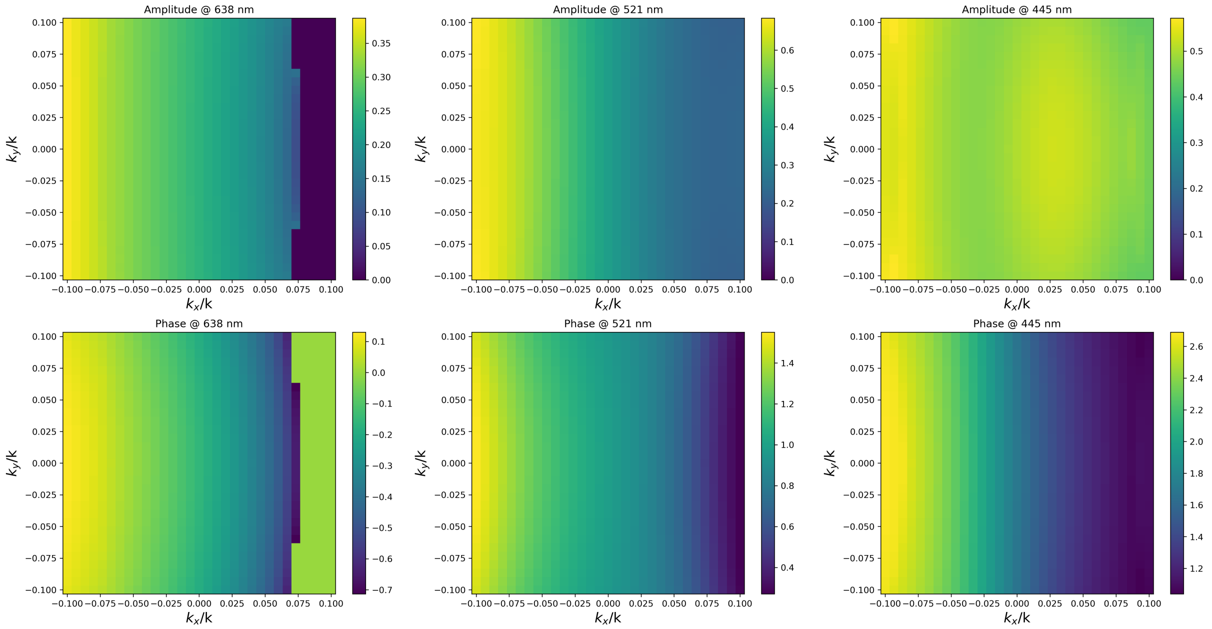

Supplementary Fig. 5 Simulated 2D transfer functions of the diffraction efficiency for conventional single-lined gratings. The amplitude (top) and phase (bottom) of the complex-amplitude transfer functions for the red (left), green (middle), and blue (right) are presented.

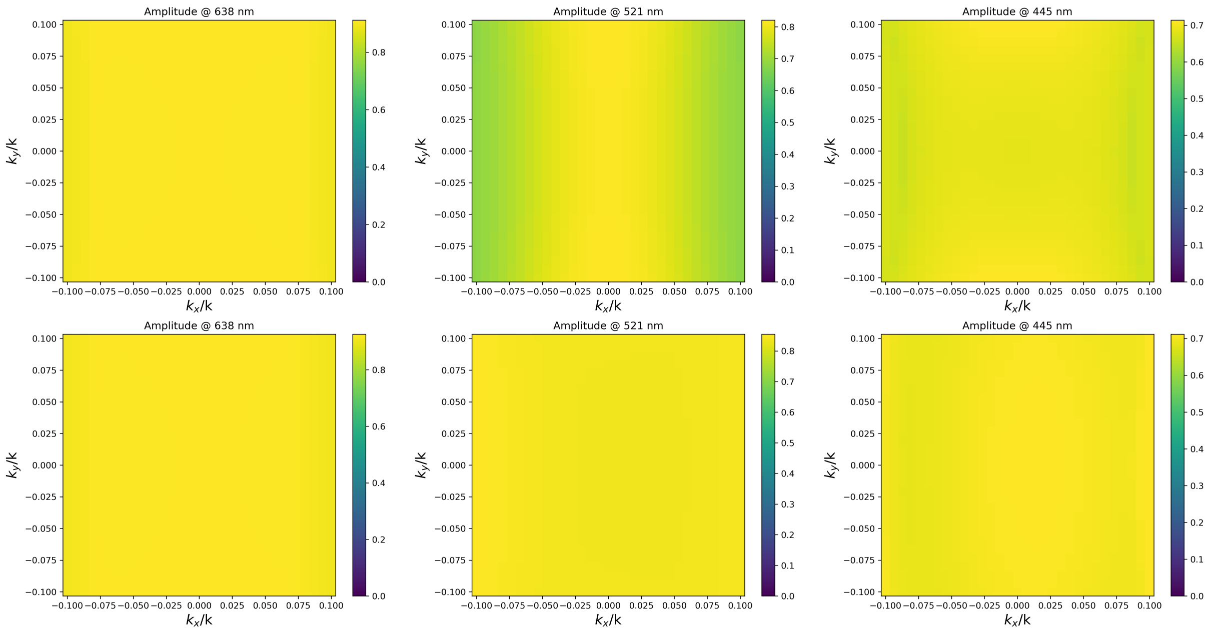

Supplementary Fig. 6 Simulated 2D transfer functions of the see-through efficiency. The amplitude of the complex-amplitude transfer functions is presented for conventional single-lined gratings (top) and our inverse-designed metasurfaces (bottom) at the red (left), green (middle), and blue (right) wavelengths.

Definition and discussion of angular uniformity

This subsection provides more detailed definitions and discussions of angular sensitivity and uniformity of our metasurfaces. The angular sensitivity in this context refers to how much the diffraction efficiency and phase delay (i.e., the transfer function) changes when light with a specific wavelength is incident on the metasurface gratings as the angle of incidence changes. For example, nanophotonic devices including metasurfaces diffract light with high efficiency for normal incidence but may have lower diffraction efficiency for oblique incidence, and this can lead to performance degradation such as reduced image quality or vignetting in imaging/display applications, such as ours, which consider a wide range of incident angles. Most prior works on metasurface gratings have focused on maximizing diffraction efficiency for a specific angle, usually normal incidence, but in our work, we optimized the metasurface gratings to have high diffraction efficiencies for both normal and oblique angles to achieve invariance of diffraction efficiency to the angle of incidence. Figure 2f shows the transfer function in the one-dimensional domain. There, we see how much the transfer function changes depending on the angle of incidence with a comparison between normal gratings and our inverse-designed metasurface gratings, which shows ours has a more uniform transfer function with a smaller variance over the incident angles at the RGB wavelengths. Furthermore, we provide an evaluation metric called "uniformity" to quantitatively evaluate the invariance of the transfer function with respect to the angle of incidence for each color, which is defined as the ratio of the minimum and maximum amplitudes within the viewing angle range. As represented in Figure 2g, the inverse-designed metasurface has high uniformities of 61.7%, 91.2%, and 98.3% for red, green, and blue color channels, respectively, whereas conventional gratings achieve much lower uniformities of 58.9%, 47.7%, and 88.8%, showing that our inverse-designed all-glass metasurface couplers provide better angular uniformity and higher see-through efficiency in the entire visible range than conventional gratings.

Intuitively, the reason why our inverse-designed metasurface is more "angle" independent than conventional gratings is that ours is able to utilize more diverse electromagnetic resonance modes inside the nanostructures. For example, the two gratings composing our double-lined metasurface gratings can produce different electromagnetic resonances, and the two can reinforce each other, improving diffraction efficiency or reducing angular dependency through constructive interferences to each other. Additionally, while our inverse-designed metasurface is composed of a high-index glass material with high transparency and sufficient light–matter interactions to manipulate light inside the double-lined nanostructures, in the future, it may be possible to enhance the light–matter interaction further by using higher refractive index dielectric materials or plasmonic materials.[13]

Discussion on metasurface design and diffraction efficiency

For our metasurface, the diffraction efficiency of red is slightly behind the efficiencies of green and blue. To improve diffraction efficiency for a specific wavelength, we could consider designing nanophotonic grating structures that achieve stronger electromagnetic resonances for the desired wavelength. In the limit, by designing a dielectric nanostructure to have both electric and magnetic dipole resonances of equal intensity for a specific wavelength, a metasurface with maximized diffraction efficiency, called a Huygens' metasurface, can be designed.[14] Structural perturbations in nanostructures to engineer spectral resonances could be another solution to further engineer the diffraction efficiency of our metasurface.[15] These approaches could be applied to push up the diffraction efficiency of red but would come at the cost of reducing the efficiency of other colors.

There are other potential directions to improve the diffraction efficiency across all three colors with further development of the metasurface design or material composition. For example, since higher refractive index materials can have stronger light-matter interactions to produce higher diffraction efficiency, we can consider other dielectric materials such as poly-Si which has a much higher refractive index ($n \sim 3.5$) than our high-index glass material ($n \sim 1.8$) or typical glasses ($n \sim 1.5$). However, silicon has absorption loss for visible light, leading to reduced see-through efficiency, which is a common phenomenon for materials with a high refractive index in the visible range.

For comparison, we designed and numerically analyzed metasurface gratings with three different materials: typical glass (SiO_2), our high-index glass (SCHOTT SF6), and poly-Si. Supplementary Figure 7 shows amplitude parametric designs with the amplitude of diffracted light for our target RGB wavelengths for different height and width configurations of the metasurface grating. Consequently, we can find that the silicon metasurface grating achieves over 30% of the diffraction efficiency while our high-index glass gratings and typical glass gratings have 10% and 1%, respectively. Notably, we see the largest increase in diffraction efficiency for red. The typical glass, high index glass, and silicon metasurface gratings achieve 1.64%, 6.86%, and 42.95% diffraction efficiency for red respectively. Therefore, it can be concluded that at the cost of reduced see-through efficiency we can enable higher diffraction efficiency, especially for red, by increasing the refractive index of the metasurface structure. As discussed in the Supplementary subsection "Further improvements in field of view", recent studies on nanophotonics and metasurfaces have shown that refractive index engineering with composite materials or nanophotonic structures can be achieved. In the future, we believe that this material engineering could enable the diffraction efficiency of metasurface gratings to be improved without sacrificing see-through efficiency.

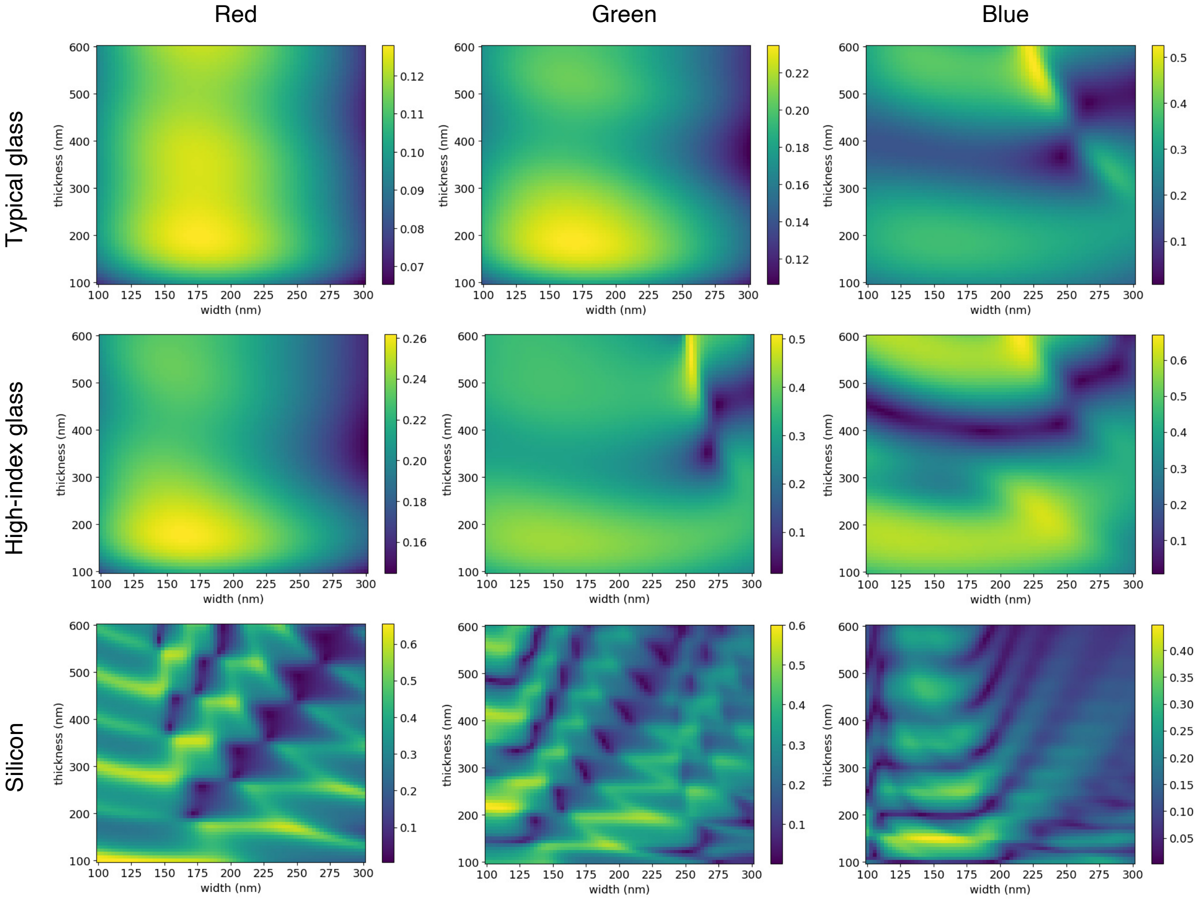

Supplementary Fig. 7 Simulation results on metasurface gratings with three different materials, including typical glass, our high-index glass, and silicon. The silicon metasurface grating can achieve over 30% diffraction efficiency while our high-index glass gratings and typical glass gratings have 10% and 1%, respectively.

Supplementary Note 3: Additional details on fabrication

The design and geometry parameters of the metasurface waveguide system is illustrated in Supplementary Figure 8a. The location and dimensions of the metasurface in- and out-coupler are designed to transfer the holographic wavefront from the in-coupler to the out-coupler for the red, green, and blue wavelengths. The dimensions of metasurfaces are 6.5 mm by 6.5 mm for the in-coupler and 6.5 mm by 6.92 mm for the out-coupler. The prototype sample is shown in Supplementary Figure 8b. The fabrication process of the metasurface waveguide system is described in the Methods of the main text and illustrated in Supplementary Figure 9.

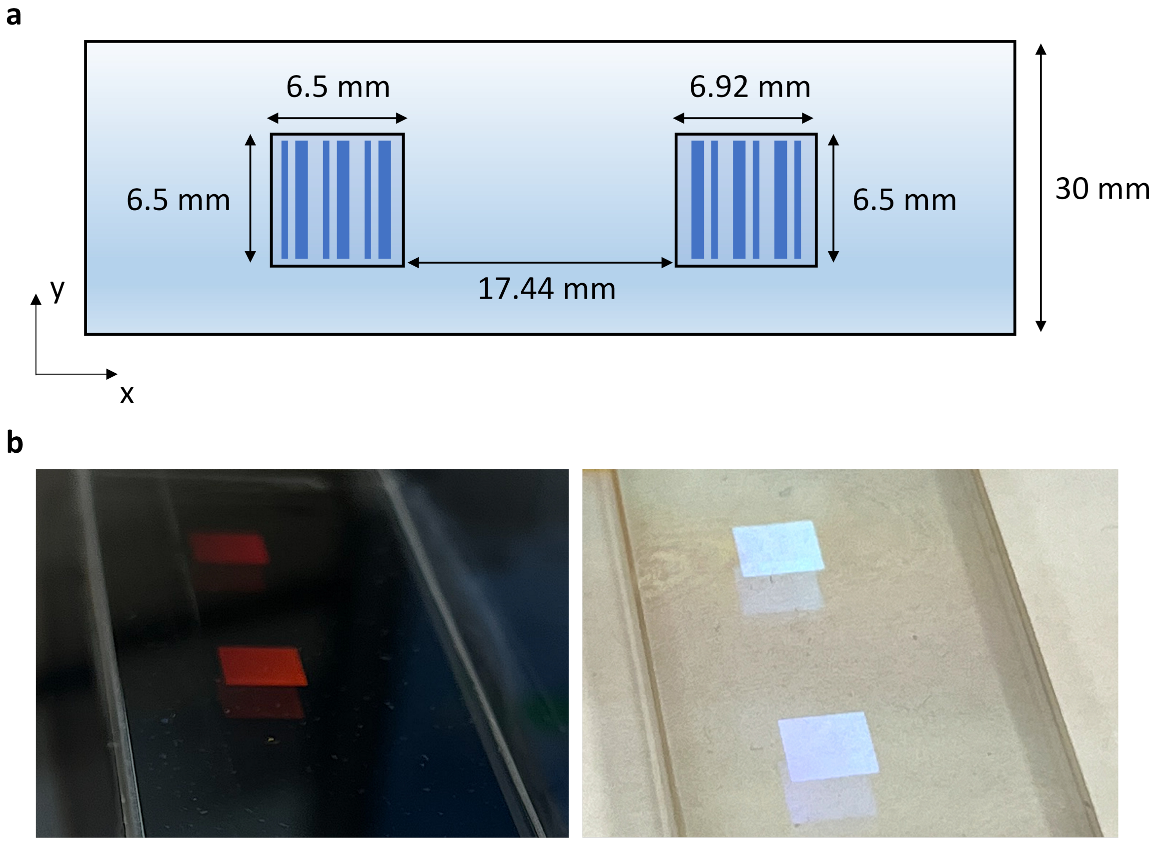

Supplementary Fig. 8 Design of the proposed metasurface waveguide system. (a) Top view of the waveguide system, illustrating key dimensions and distances within the structure. The image includes the positions and dimensions of the metasurface in- and out-couplers on the glass substrate. This schematic view provides a comprehensive understanding of the overall system design. (b) Corresponding images of the fabricated sample as captured by a camera. The image showcases the real-world implementation of the system, validating the design specifications.

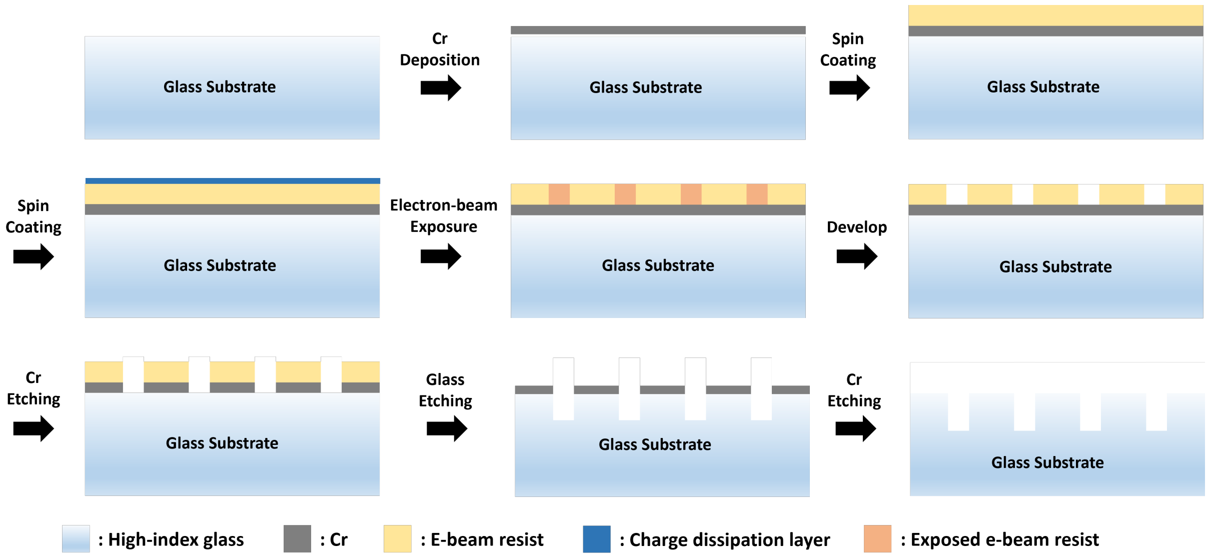

Supplementary Fig. 9 Fabrication process for the metasurface waveguide system as detailed in the Methods section. The schematic elucidates the sequential steps of the procedure: the initial preparation of a custom glass substrate with a high refractive index, followed by coating the substrate with a 30 nm thick layer of Cr film using an electron beam evaporator. Subsequent steps include electron beam lithography, which encompasses spin coating of positive electron beam resist (950 PMMA a4) and charge dissipation layer (e-spacer), electron beam exposure, resist development, and cleaning. Multiple dry etching procedures are used to transfer the metasurface patterns of the PMMA layer to the high-index glass substrate: (1) Cr etching with a patterned PMMA mask using inductively coupled plasma reactive ion etching (ICP-RIE) with a gas mixture of Cl_2, O_2, and Ar. (2) Glass etching with a patterned Cr mask using reactive ion etching (RIE) with a gas mixture of CHF_3, CF_4, and Ar. Due to the low etching rate of the lead-containing high-index glass, this etching process requires a very long etching time, so the entire process consists of repeated cycles of etching and cooling (2 min etching and 1 min cooling, 12 times) to minimize thermal damage to the Cr mask. The cooling process includes N_2 gas exposure on the sample and Helium backside cooling. (3) Etching of the remaining Cr mask using ICP-RIE again. The progression of images in this figure provides a visual comprehension of the intricate and sequential fabrication process of the metasurface waveguide system.

Transparency and rainbow effect of the metasurface gratings

For evaluation purposes, we fabricated additional metasurface samples composed of the same high-index glass with our designed metasurface grating as shown in Supplementary Figure 10. As shown in Supplementary Figure 10a, the metasurface samples are highly transparent with high see-through efficiency without significant color distortion. We also measure the see-through efficiency in the entire visible range and the diffraction efficiency at the target RGB wavelengths using the measurement setup illustrated in Supplementary Figure 12. The measured results are represented in Figures 2e and 2f compared with the simulation results, showing high see-through efficiency in the entire visible range.

Diffraction gratings, including conventional surface relief gratings and also our metasurface gratings, diffract/reflect not only the displayed signal but, to a much lesser extent, also external light from the physical scene in front of it. The diffraction or reflection of external light may, in rare instances, cause a rainbow effect. This effect is inherent to all diffraction gratings and, despite the potential artifacts this effect causes, diffraction grating-based waveguides are the industry standard for OST-AR displays. Indeed, our approach is less prone to this effect than the waveguides used by commercial solutions. Metasurfaces offer unique abilities in this context by tuning gratings to increase see-through efficiency and decrease reflectance or other diffraction orders, which is an interesting avenue for future work. To be more specific, one advantage of our metasurface gratings is that the transmission from the glass substrate to air is free of diffraction modes due to the subwavelength grating periodicity. For example, the period of our metasurface grating is 384 nm, which is smaller than the wavelength of visible light in air, so light transmitted through the metasurface, from glass substrate to air, cannot be coupled into diffraction modes in air while the metasurface can still diffract the visible light in the glass substrate with a high refractive index. This allows selective control of the diffraction mode depending on the propagating material (e.g., air or glass). For this reason, our gratings have a smaller rainbow effect by the absence of transmitted diffraction orders through the metasurface while there are still small amounts of rainbow effect from direct reflections from the sample.

For an experimental demonstration of the rainbow effect, real-scene images with and without the metasurface samples are captured to demonstrate transparency and the rainbow effect under sunlight. As shown in Supplementary Figure 11, our inverse-designed all-glass metasurfaces still provides a very clear image without significant color distortion.

There are orthogonal lines of work which have sought to mitigate this effect with nonlocal metasurface gratings, which have strong angular or spectral dependency.[16] The core of the nonlocal metasurface is that the metasurface grating periodicity is subwavelength, so in addition to our advantage of being able to selectively control the diffraction order, we can further reduce the rainbow effect by providing additional angular/spectral selective properties to the grating nanostructures. In the future, it would be interesting to integrate those techniques into our metasurface design.

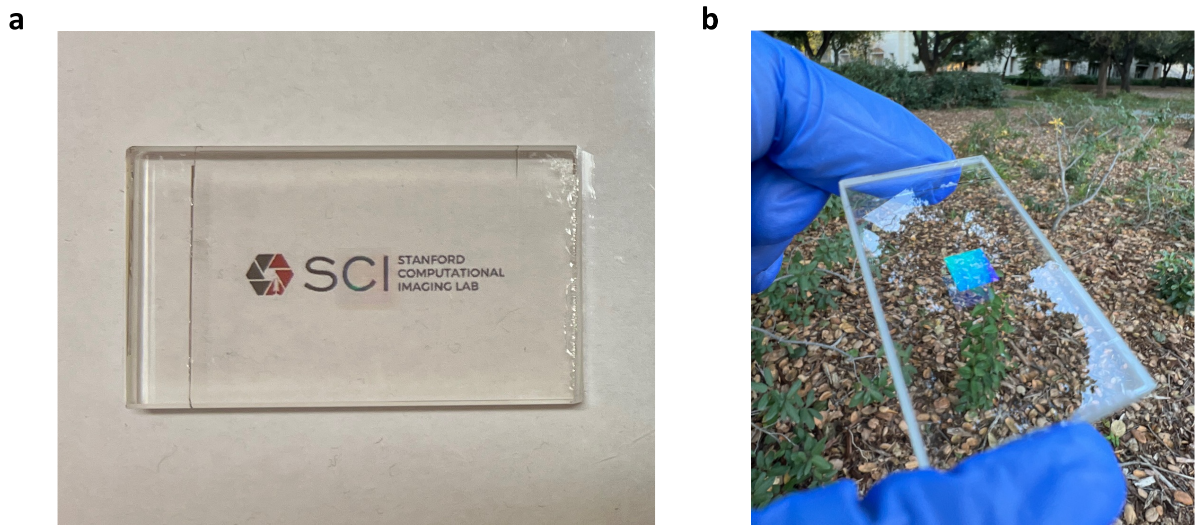

Supplementary Fig. 10 Captured images of a fabricated metasurface sample. The metasurface sample is composed of a high-index glass substrate and a single metasurface grating with a dimension of 5 mm by 5 mm. The metasurface samples are highly transparent with high see-through efficiency, as shown in (a), but for particular light source locations and viewing directions, the direct reflections of ambient light show a rainbow effect common for all grating-based AR waveguides, as shown in (b). The images were captured (a) inside the laboratory and (b) outside during the day.

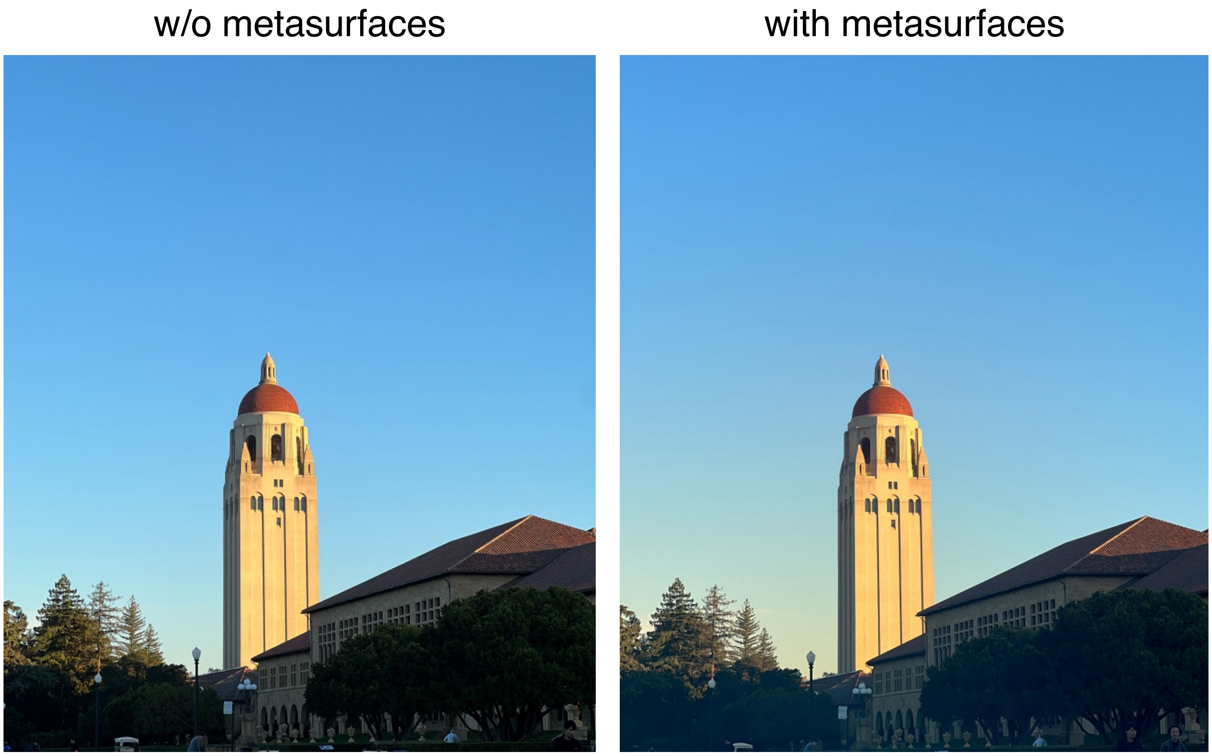

Supplementary Fig. 11 Captured real-scene images (left) without or (right) with the metasurface sample. The images were taken outside during the day using a smartphone camera. The image without the metasurface sample was captured directly with the camera, and the image with the metasurface sample was captured through the metasurface sample in front of the camera. The capture image shows very high transparency under sunlight during the day and demonstrates that the proposed metasurface waveguide system is suitable for AR applications.

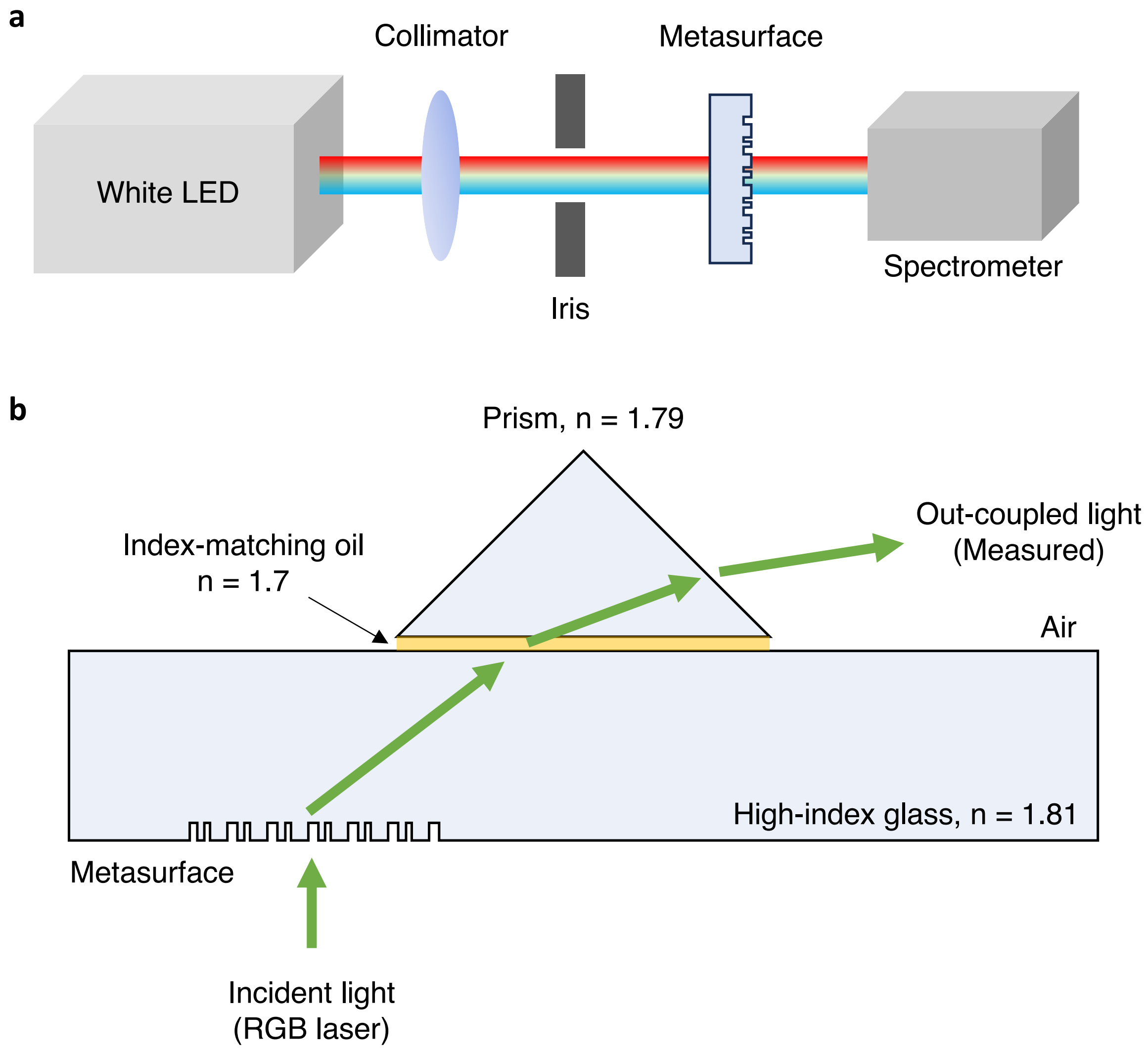

Supplementary Fig. 12 Schematic diagram showing the measurement setup for the see-through efficiency and transfer function of the inverse-designed metasurfaces. (a) See-through efficiency measurement using white LED light source and spectrometer. (b) Transfer function measurement using laser light source and prism attachment. Note that it is not possible to directly measure the signal from the metasurface sample because the diffraction angle of our inverse-designed metasurfaces is very high and the diffracted light is trapped inside the waveguide system by total internal reflections, so a high refractive index prism ($n = 1.79$, Thorlabs) and index matching oil ($n = 1.7$, Norland) are used to out-couple the light inside the waveguide. The input laser source is a fiber-coupled laser with the target RGB wavelengths.

Supplementary Note 4: Additional details on waveguide model

Deriving the Frequency-Dependent Waveguide Transfer Function

In our waveguide model, the transfer function, H_{wg}, models the full phenomena within the waveguide as a set of complex-valued frequency-domain coefficients. Firstly, these coefficients include the frequency-dependent coupling efficiencies of our designed gratings as computed by RCWA simulations, H_{out} and H_{in}. Secondly, the coefficients include the phase delay, $\phi_{reflect}$, produced by the N_{TIR} total internal reflections within the waveguide.

$$\phi_{reflect} = -2 \cdot N_{TIR} \cdot \arctan\left(\frac{n(\lambda)\sqrt{\lambda^2\left(\left(f_x + \frac{1}{\Lambda}\right)^2 + f_y^2\right) - 1}}{\sqrt{1 - \left(\frac{\lambda}{n(\lambda)}\left(f_x + \frac{1}{\Lambda}\right)\right)^2 - \left(\frac{\lambda}{n(\lambda)} f_y\right)^2}} \right)$$

Next, the coefficients include a phase delay based on the optical path length traversed by each frequency within the waveguide:

$$\phi_{prop} = \frac{2\pi n(\lambda)}{\lambda} d_{wg}\left(N_{TIR} + 1\right)\sqrt{1 - \left(\frac{\lambda}{n(\lambda)} f_x + \frac{\lambda}{n(\lambda)\Lambda}\right)^2 - \left(\frac{\lambda}{n(\lambda)} f_y\right)^2}$$

Lastly, based on the shifting property of the Fourier Transform, the transfer function includes a frequency-dependent phase ramp, $\phi_{translate}$ to translate the coordinates of the wavefront from the in-coupler to the out-coupler (where t_{wg} is the distance between the centers of the couplers):

$$\phi_{translate} = 2\pi f_x t_{wg}$$

Putting together the coupling efficiencies, the total internal reflections, the optical path length propagation, and the coordinate translation, we get the full expression for the frequency-dependent coefficients of the transfer function through the waveguide:

$$H_{wg} = H_{out} H_{in} e^{i\left(\phi_{reflect} + \phi_{prop} + \phi_{translate}\right)}$$

Bandwidth considerations of waveguide model

One major consideration for implementing our proposed image formation model is the amount of sampling required to model the wavefronts without aliasing and the padding required to model the propagations without circular convolutions.

To determine the amount of sampling required to avoid aliasing, we can look at the angular ranges spanned by the wavefronts at different points within our propagation pipeline since this directly corresponds to the frequencies present within the wavefront. To avoid aliasing for the wavefront at the in-coupler, we reconstruct the wavefront based on the Nyquist-Shannon sampling theorem to be able to model the maximum deflection frequency of the SLM added to the maximum frequency of the illumination wavefront. After constructing the wavefront at this high sampling, this wavefront can be downsampled with anti-aliasing to model only the angular frequencies that correspond to the optimized FOV of the display. This sampling methodology also enables our model to explicitly simulate the contribution of higher diffraction orders to the optimized FOV of our display. While the zero-order undiffracted light is not explicitly modeled with our physical waveguide model, it can be characterized at this simulation bandwidth with our learned physical waveguide model.

When this wavefront is propagated through the waveguide, the wavefront must be padded to avoid circular convolution. To do this, we pad the wavefront to twice the original area and use the same methodology as the band-limited angular spectrum method (ASM)[17] to zero out frequencies in the transfer function that would be aliased. Here we also zero out frequencies that would propagate to areas outside our optimized FOV.

To model the free-space propagation in our system, we apply a diverging wavefront, corresponding to a simulated imaging lens, that cancels out the focal power of the incident converging wavefront. Then we use ASM propagation to propagate to the desired re-imaged target distances. When we apply this imaging lens, we must again upsample the wavefront to avoid aliasing the imaging lens, but we can again antialias blur and downsample the wavefront after the lens before propagating to the target planes. Additionally, since we already blurred out frequencies that would propagate to areas outside our optimized FOV, we do not need to pad our wavefront before propagating to all the target planes. This explicit integration of a free-space propagation model enables our architecture to accurately model long propagation distances.

Our implementation of our learned physical waveguide model, which is built on this physical model, is not optimized for speed and is trained on eight NVIDIA RTX A6000 GPUs for a day. This training time could be significantly reduced by optimizing our model using a custom CUDA implementation. We did not focus on optimizing this part of the pipeline, because it only needs to be done once, as a one-time, post-fabrication calibration routine. During runtime, holograms are synthesized using standard phase-retrieval-based computer-generated holography algorithms that invert our learned physical waveguide model. In our current implementation, 2D hologram generation takes 10 minutes per phase pattern, and 3D hologram generation takes 15 minutes per phase pattern. However, prior works have demonstrated that real-time phase

synthesis networks can be trained to invert forward models for 2D and 3D computer-generated holography.[1,18,19]

Ablating the model performance

In Supplementary Table 1, we quantitatively evaluate the accuracy of different wave propagation models on predicting the output of our holographic AR system. For baselines, we compare our waveguide models against the ASM model for free-space propagation as well as a recent state-of-the-art learned propagation model[3] designed around free-space propagation. Our physical waveguide model with no learned parameters performs slightly better than free-space propagation. Once we fit the spatially varying efficiencies at the gratings, the performance of our model improves substantially. Our full learned physical waveguide model with CNNs and the parameterized gratings performs the best, and significantly outperforms the free-space propagation models. Additionally, while the learned free-space model can fit reasonably well for our captured dataset, it fails to generalize well enough to produce high quality images during inference since it is built around an incorrect physical model. This poor inference performance for the learned free-space model is shown in additional experimental results in this Supplement.

Supplementary Table 1 Performance comparison of different wave propagation models. All models are evaluated on 4 planes. Peak signal-to-noise ratio (PSNR) is evaluated for training and test sets.

Models	Train	Test
Free-Space Model	23.50 dB	23.12 dB
Learned Free-Space Model[3]	33.07 dB	32.49 dB
Physical Waveguide Model	24.80 dB	24.25 dB
+ a_{IC} + a_{OC}	30.52 dB	29.54 dB
+ a_{IC} + a_{OC} + CNN_{IC}+ $\text{CNN}_{\text{target}}$	**36.51 dB**	**35.75 dB**

Supplementary Note 5: Additional experimental results

Additional 2D Comparison of Wave Propagation Models

Supplementary Figure 13 is an additional 2D comparison that highlights the benefits of our proposed propagation model that pairs physical waveguide modelling with learned components.

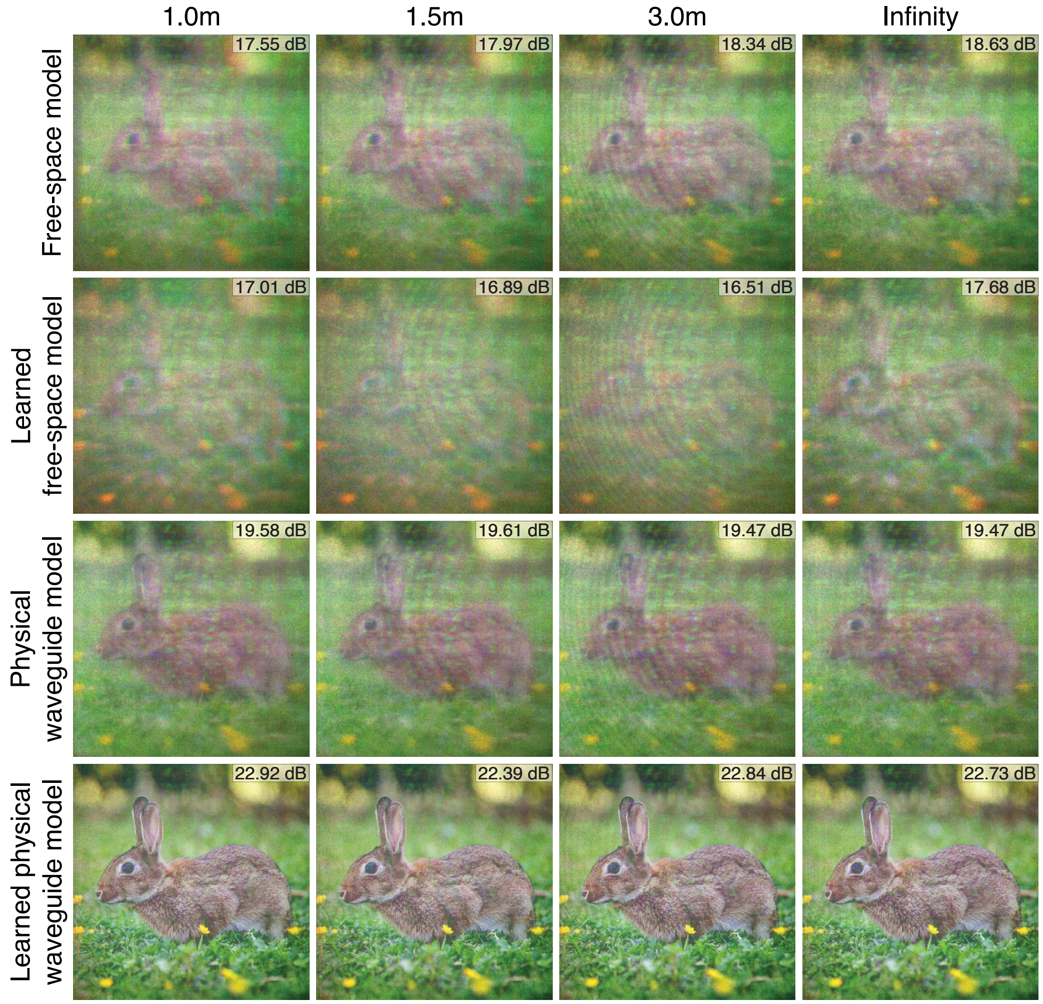

Supplementary Fig. 13 2D holograms optimized for different distances with different models of propagation. The image quality with the free-space propagation model is poor especially at closer virtual distances. The learned free-space model[3] does not improve image quality since the model overfits to the training dataset without learning the actual physical propagation through the waveguide. Across all distances, optimizing through the physical waveguide model produces better image quality than optimizing through the free-space propagation models. After adding our proposed learned parameters to this correct physical model of the waveguide, the resulting image quality is substantially further improved.

Additional 3D Comparison of Wave Propagation Models

Supplementary Figure 14 is an additional 3D comparison that similarly highlights the benefits of our proposed pairing of a physical waveguide model with learned components.

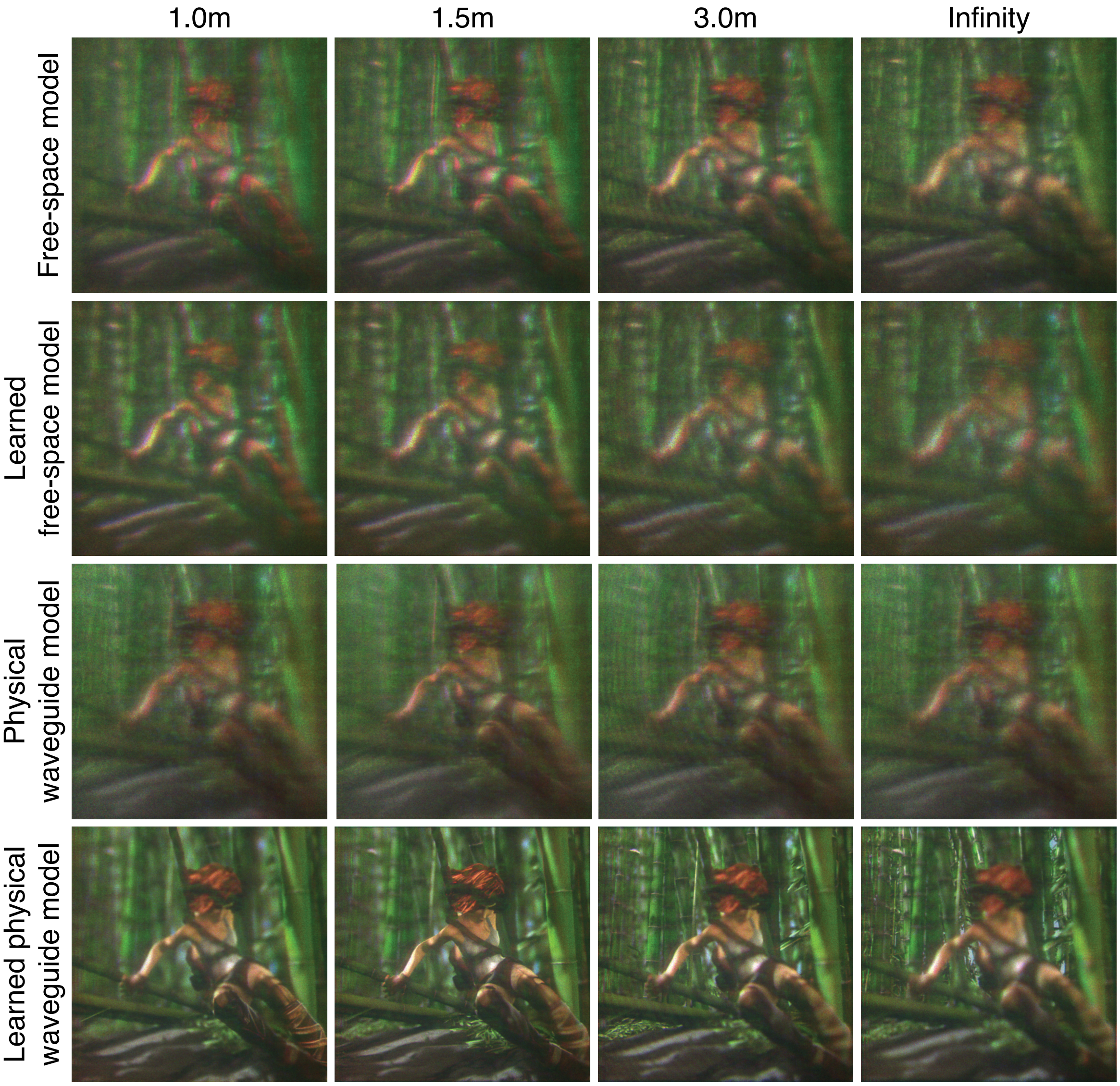

Supplementary Fig. 14 3D holograms optimized with different models of propagation. These show similar patterns to the 2D captures. The image quality with the free-space propagation models are poor with and without learning. The image quality with the physical waveguide model is slightly better, and the image quality with our proposed learned physical waveguide model is best. The target scene is from the Durian Open Movie project (© copyright Blender Foundation, www.sintel.org) under a Creative Commons licence (https://creativecommons.org/licenses/by/3.0/).

Resolution Chart Comparison Between Wave Propagation Models

Supplementary Figure 15 illustrates the improvement in sharpness provided by our proposed model. Producing sharp content with holography requires distant pixels to interfere accurately, so precise modeling is required. Our learned parameters enable this.

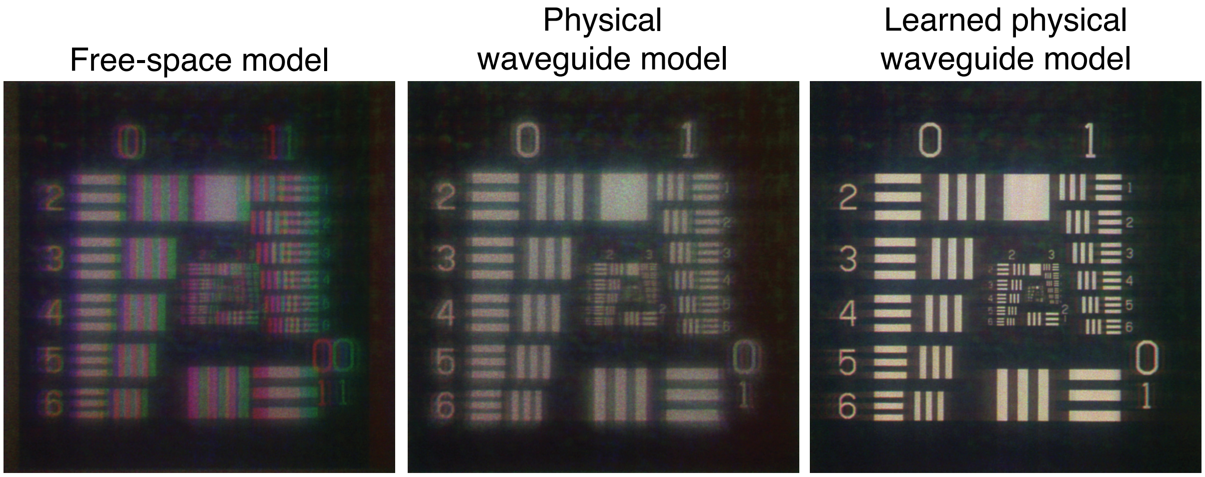

Supplementary Fig. 15 2D comparison between wave propagation models with a resolution chart target figure. This comparison further illustrates the benefits of the physical waveguide model and our proposed learned physical waveguide model.

Comparison between Different Levels of Time Multiplexing

Supplementary Figure 16 experimentally illustrates the benefits of time multiplexing for producing smooth defocus behavior.

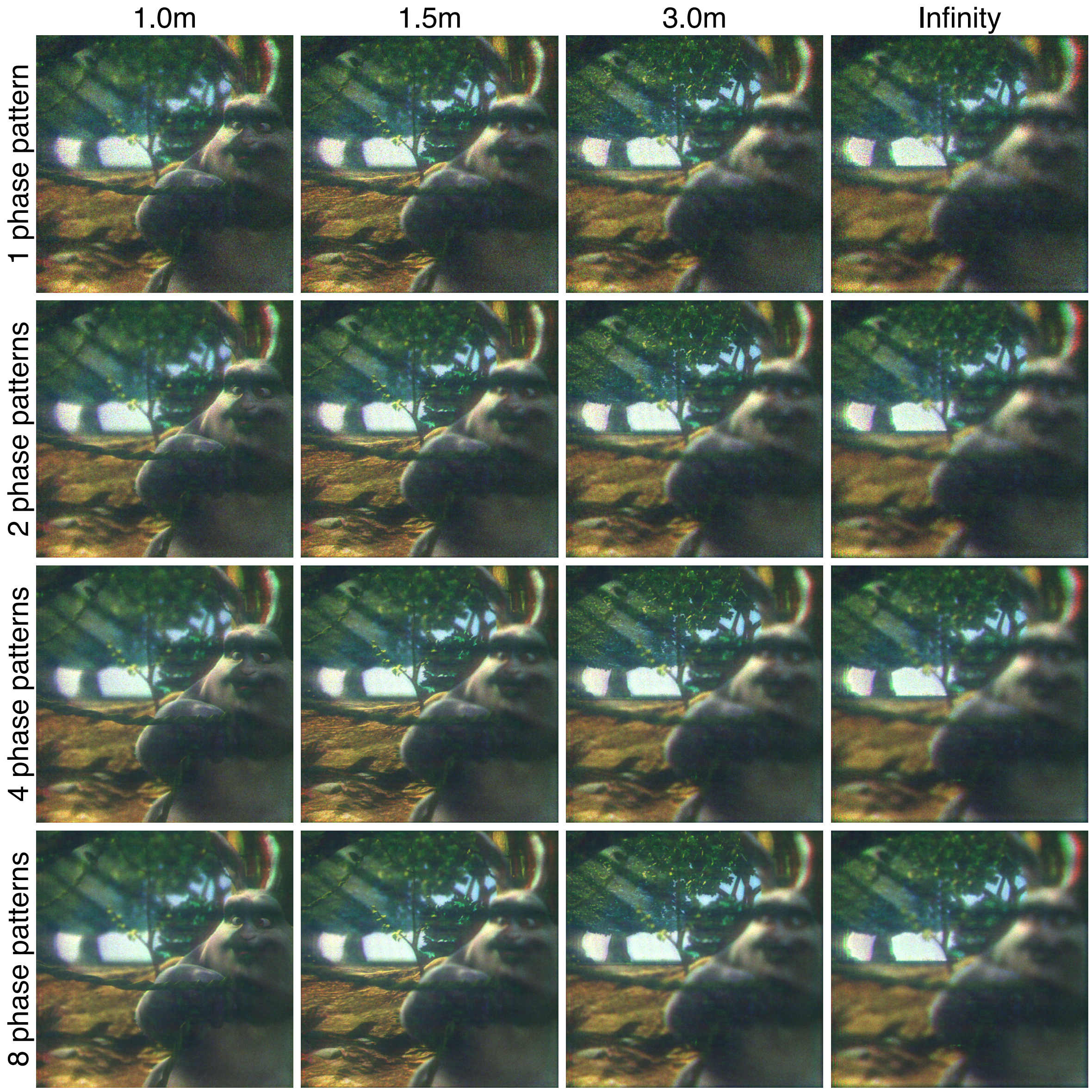

Supplementary Fig. 16 3D holograms produced with different numbers of time-multiplexed phase patterns. When only a single phase pattern is used, the hologram presents a full view of the 3D scene. As more phase patterns are time multiplexed, the resulting partially coherent hologram has reduced speckle and more natural (i.e., incoherent) defocus blur. This echoes the conclusions in prior work.[3] The target scene is provided by peach.blender.org (© 2008, Blender Foundation) under a Creative Commons licence (https://creativecommons.org/licenses/by/3.0/).

Comparison between Views from across the Eyebox

Supplementary Figure 17 shows images seen through the waveguide with different amounts of horizontal camera translation ranging from -2 mm to 2 mm. From these results, it can be seen that the benefits of our learned physical waveguide model are not overfit to a particular camera position and enable improvements to image quality across the eyebox.

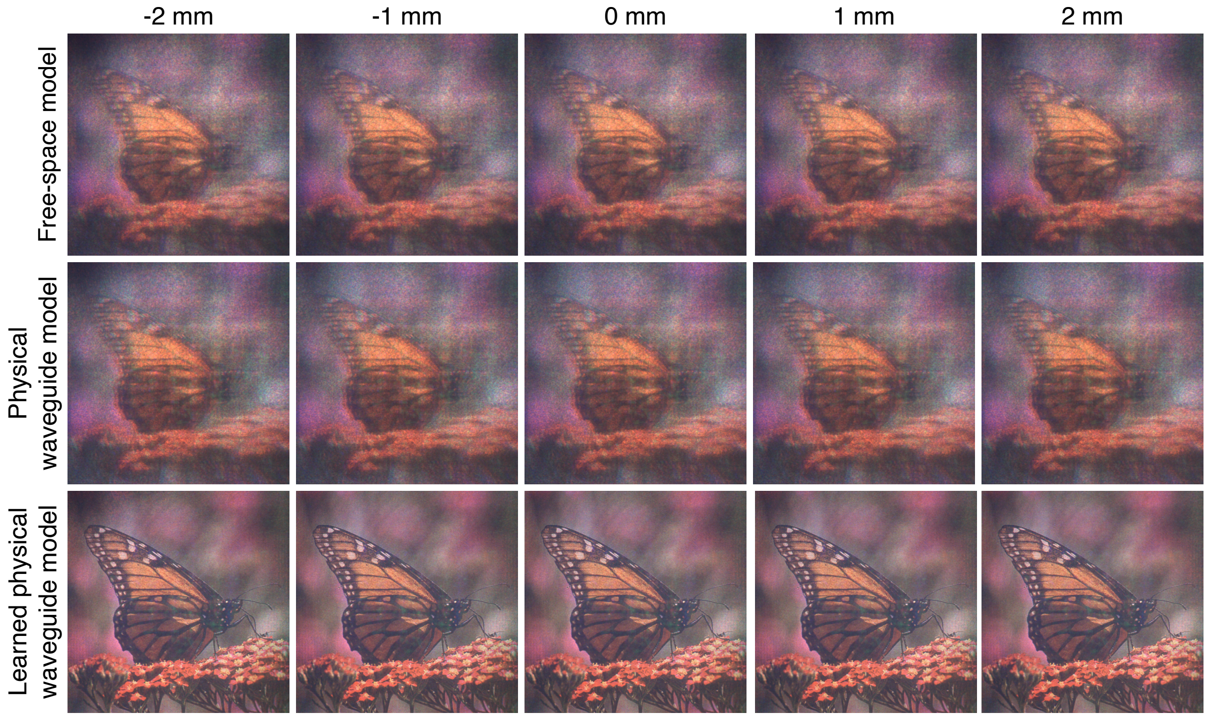

Supplementary Fig. 17 Views captured with different camera translations across our eyebox. The high image quality with our learned physical waveguide model is maintained for different camera translations.

Laser-synchronized Video Holograms

To demonstrate the full-color capabilities of our system, we have captured the view of laser-synchronized holograms from our display. For these video results, all three colors are captured together in the same exposures without any digital post processing. The synchronization for these results is performed by operating our Holoeye SLM in color-field sequential (CFS) mode. In this mode, the SLM rapidly switches between phase patterns for each of the primary colors at 180 Hz, and the driver board of the SLM outputs three illumination synchronization signals which we use as an analog enable for our three laser wavelengths. With this configuration, illustrated in Supplementary Figure 18, our FISBA READYBeam pulses the colors of illumination in-sync with the phase patterns displayed on the SLM.

In our Supplementary video, we include multiple video results captured with laser-syn-

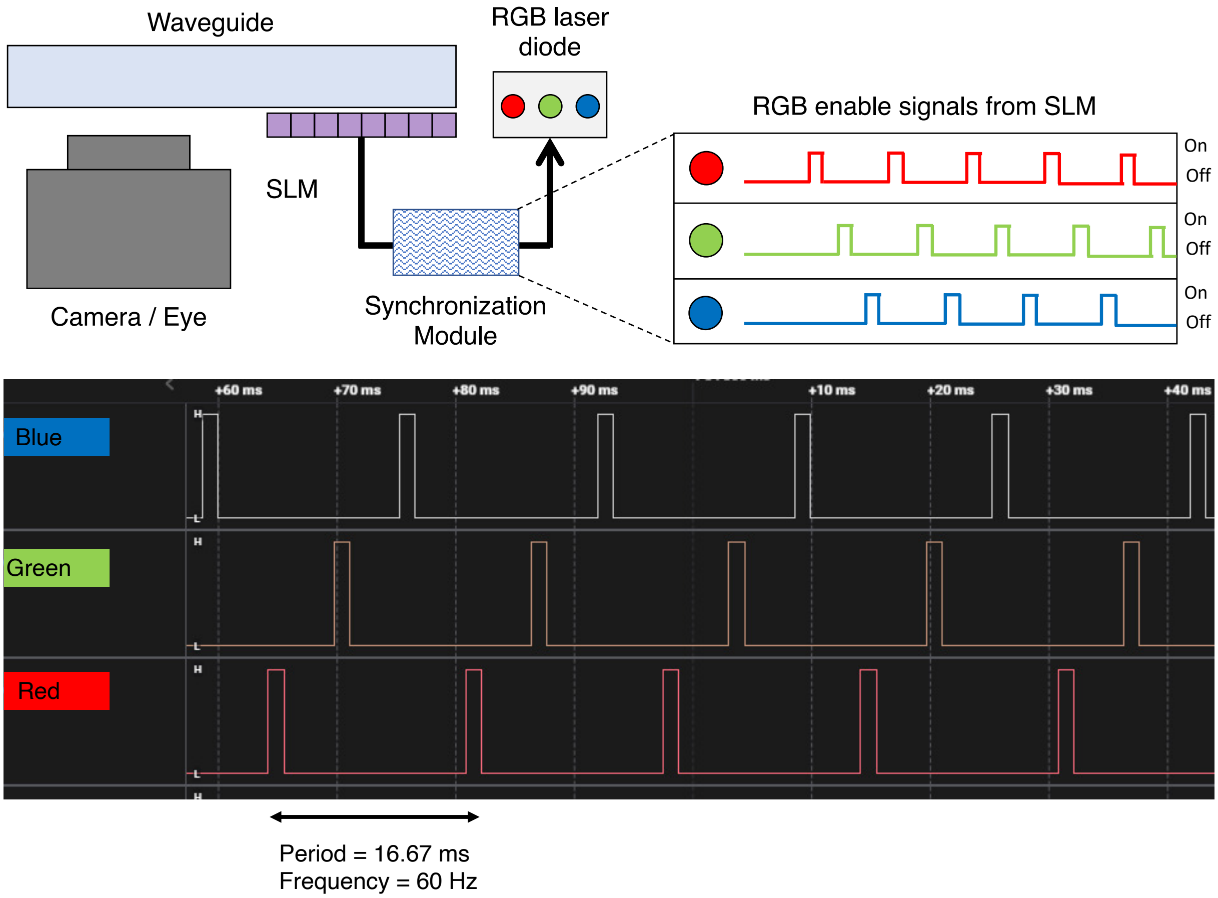

Supplementary Fig. 18 (Top) Diagram of laser-synchronization configuration. The SLM driver outputs RGB enable signals to coordinate the timing of the illumination and the displayed holograms. (Bottom) RGB enable signals from our HOLOEYE LETO-3 SLM measured using a Saleae Logic Pro 16.

chronized holograms. First, we show a clip of laser-synchronized 2D video results. In these results, the image quality with the free-space propagation model is poor with low resolution and vignetting. The image quality with the physical waveguide model slightly improves the vignetting, but is still poor due to a less accurate look-up table when the SLM is operating in CFS mode. With our learned physical waveguide model, these aberrations are overcome, and high image quality is produced. The target video is provided by peach.blender.org (© 2008, Blender Foundation) under a Creative Commons licence (https://creativecommons.org/licenses/by/3.0/). We next include a comparison of laser-synchronized 3D video results. These results exhibit similar behavior; our full learned physical waveguide model can be used to generate high-quality holograms. For this 3D video clip, we also show the first phase modulation pattern for the green channel for each frame. When the 3D video is paused and the camera is changing focus, the displayed hologram does not respond to the camera's focus illustrating how the holograms simultaneously produce the full 3D scene. The target video is from the Durian Open Movie project (© copyright Blender Foundation, www.sintel.org) under a Creative Commons licence (https://creativecommons.org/licenses/by/3.0/). We also include 2D and 3D AR results that demonstrate the high AR image quality with our learned physical waveguide model. In the 2D AR scene, a real world koala is scanned by a virtual HUD that is produced by our holographic display. In the 3D AR scene, virtual toys, including a bike, a cube, and a robot, are positioned around a real world action figure and duck. Our display provides the correct scale of blur on virtual content for human pupil diameters up to the 5.9 mm eyebox size. As the pupil or camera aperture grows beyond this size, the blur of the real-world objects continues to grow while the blur of virtual content does not. Since we capture our results with a programmable focus Canon lens that has a larger aperture, the blur on the real world content appears large, while the blur of the virtual content is at the scale that would be perceived through a 5.9 mm pupil. This produces the deviation in defocus behavior for virtual and real-world content in our AR results.

Supplementary References

[1] Peng, Y., Choi, S., Padmanaban, N. & Wetzstein, G. Neural holography with camera-in-the-loop training. *ACM Transactions on Graphics (TOG)* **39**, 1–14 (2020).

[2] Choi, S., Gopakumar, M., Peng, Y., Kim, J. & Wetzstein, G. Neural 3d holography: Learning accurate wave propagation models for 3d holographic virtual and augmented reality displays. *ACM Trans. Graph. (SIGGRAPH Asia)* (2021).

[3] Choi, S. *et al.* Time-multiplexed neural holography: A flexible framework for holographic near-eye displays with fast heavily-quantized spatial light modulators. In *Proceedings of the ACM SIGGRAPH*, 1–8 (2022).

[4] Kim, J. *et al.* Holographic glasses for virtual reality. In *Proceedings of the ACM SIGGRAPH*, 1–8 (2022).

[5] Yu, N. *et al.* Light propagation with phase discontinuities: generalized laws of reflection and refraction. *science* **334**, 333–337 (2011).

[6] Lee, G.-Y. *et al.* Metasurface eyepiece for augmented reality. *Nature Communications* **9** (2018).

[7] Hwang, C.-S. *et al.* 21-2: Invited paper: 1um pixel pitch spatial light modulator panel for digital holography. *SID Symposium Digest of Technical Papers* **51**, 297–300 (2020).

[8] Park, J. *et al.* All-solid-state spatial light modulator with independent phase and amplitude control for three-dimensional lidar applications. *Nature nanotechnology* **16**, 69–76 (2021).

[9] Li, Q. *et al.* A purcell-enabled monolayer semiconductor free-space optical modulator. *Nature Photonics* **17**, 897–903 (2023).

[10] Yoon, G., Kim, K., Huh, D., Lee, H. & Rho, J. Single-step manufacturing of hierarchical dielectric metalens in the visible. *Nature Communications* **11**, 2268 (2020).

[11] Kim, J. *et al.* Scalable manufacturing of high-index atomic layer–polymer hybrid metasurfaces for metaphotonics in the visible. *Nature Materials* **22**, 474–481 (2023).

[12] Kim, C. & Lee, B. Torcwa: Gpu-accelerated fourier modal method and gradient-based optimization for metasurface design. *Computer Physics Communications* **282**, 108552 (2023).

[13] Lin, D. *et al.* Optical metasurfaces for high angle steering at visible wavelengths. *Scientific reports* **7**, 2286 (2017).

[14] Decker, M. *et al.* High-efficiency dielectric huygens' surfaces. *Advanced Optical Materials* **3**, 813–820 (2015).

[15] Lawrence, M. *et al.* High quality factor phase gradient metasurfaces. *Nature Nanotechnology* **15**, 956–961 (2020).

[16] Song, J.-H., van de Groep, J., Kim, S. J. & Brongersma, M. L. Non-local metasurfaces for spectrally decoupled wavefront manipulation and eye tracking. *Nature Nanotechnology* **16**, 1224–1230 (2021).

[17] Matsushima, K. & Shimobaba, T. Band-limited angular spectrum method for numerical simulation of free-space propagation in far and near fields. *Optics express* **17**, 19662–19673 (2009).

[18] Shi, L., Li, B., Kim, C., Kellnhofer, P. & Matusik, W. Towards real-time photorealistic 3d holography with deep neural networks. *Nature* **591**, 234–239 (2021).

[19] Shi, X. *et al.* Design of a dual focal-plane near-eye display using diffractive waveguides and multiple lenses. *Applied Optics* **61**, 5844–5849 (2022).
